# Is State-Wise Healthcare Budget Allocation Consistent With the Disease Burden in India? A Quinquennial Account (2015–2019)

**DOI:** 10.3389/fpubh.2022.893257

**Published:** 2022-06-28

**Authors:** Bhavani Shankara Bagepally, S. Sajith Kumar, Akhil Sasidharan

**Affiliations:** Health Technology Assessment Resource Centre, Indian Council of Medical Research-National Institute of Epidemiology, Chennai, India

**Keywords:** DALY, India, budget allocation, disease burden, healthcare

## Abstract

**Introduction:**

Evidence-based resource allocation may help to achieve immense health gains in resource-limited settings like India. Understanding healthcare expenditure and the corresponding disease burden could provide insights to plan optimal allocation of limited resources. Hence, we aimed to investigate the status and trends of state-wise healthcare budget allocation and the corresponding disease burden.

**Methods:**

We retrieved state-wise healthcare budget allocation information in India for the years 2015 to 2019. Corresponding state-wise disability-adjusted-life-year (DALY) estimates from the Global Burden of Disease, injuries, and Risk Factors Study (GBD) was used to measure disease burden. The allocated budget (in rupees) per DALY was calculated for overall, communicable, and non-communicable diseases (NCDs). Descriptive statistics, correlation and graphical representations were used to identify and evaluate the trends and relationships between state-wise health budget allocation and disease burden.

**Results:**

The allocated budget per DALY in 2019 was highest for Goa (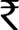 34,260 or US$ 486.66) and lowest for Bihar (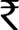 2,408 or US $ 34.20). Smaller, less populous states had higher budget allocations per DALY than larger states. Health budget allocation had an inverse relationship with infectious diseases and an identical linear relationship with NCDs. Most state-wise health budget allocations, as well as total disease burden, increased over the years except for Assam, Karnataka, and Himachal Pradesh. Also, such trends are not similar for the injuries and NCD disease burden.

**Discussion:**

The health budget allocation is variable across states as well as between infectious and NCDs. The current increase in the allocated budget is incongruent with the increasing disease burden. There is a need for rapid expansion of healthcare resource allocation guided by evidence in India.

## Introduction

India accounts for nearly one-fifth of the world population, and the level risk factors for disease as well as health status vary widely across states. Hence, achieving the Universal Health Coverage (UHC) by 2030 (1) would require a systematic understanding of state-wise health trends in each state for health financing. Since the current health expenditure in India is one of the lowest in the region, scaling up resource allocation to achieve UHC is challenging. For the effective allocation of limited resources, context-specific health economic evidence is essential. However, it is challenging to generate such evidence to inform priority setting in India, where government goals and political interests may take precedence over public health needs or disease burden. In this context, disability-adjusted life years (DALY) could provide understandable representations of disease burden for the target population along with quantification (2). The robust evidence on trends in health budget allocation and its impact on disease burden represented in and as DALY changes could be a crucial input for evidence-based policymaking at the national and state level.

India State-level Disease Burden Initiative, 2017 reported a 36 percent drop in DALY during the 1990–2016 period in India (3). The state level trend in disease burden differs highly between states like Assam, Uttar Pradesh, and Chhattisgarh having the highest rates, and Kerala and Goa in the lower end (3). The same level of variation is evident in the epidemiological transition for states like Kerala, Goa, and Tamil Nadu dominates in non-communicable diseases, whereas this dominance is relatively lower in Bihar, Jharkhand and Uttar Pradesh (3). Thus, each state in India differs in terms of its health status and morbidity pattern. Current health expenditure (% of Gross Domestic Product-GDP) reduced to 3.01% in 2019 from 3.60% in 2015 (4). The most recent trends in municipal finances in India are unavailable but according to a 2001–02 estimate, only 2.4% of the total municipal budget was allotted to health, which would account for a meager 0.02% of the 2001-02 GDP (5). According to the fifth National Family Health Survey, NFHS-5 (2019–21), households with any usual member covered under a health insurance/financing scheme increased to 41%, compared to 28.7% during the NFHS-4 (2015–16) survey (6).

In India, each state is responsible for its own population's health and thus has its own healthcare system. The federal government oversees national strategic planning, health regulation, international health, and poorer state subsidization. So, government health care spending and budgeting should be prudent. Imbalanced resource allocation, limited physical access to quality health services, and inadequate human resources for health, out-of-pocket health expenditures, health spending inflation, and behavioral factors are established factors that affect the demand for appropriate health care (7). DALYs, as a health indicator, can be linked to other indicators and, as well, be used as a proxy for understanding healthcare expenditure and even the distribution of local wealth. It can be related to reducing DALYs; fewer DALYs are better for public health and reduce healthcare spending (8). The scientific understanding of the effects of health policies and associated public healthcare budget allocation among Indian states must evolve. In a country like India, efficient allocation of scarce healthcare resources by policymakers is crucial (9). Tools such as the “Global Burden of Disease (GBD) India Compare” could assist in understanding disease burden trends across states. However, budget allocation varies by state and is usually not based on disease burden estimates. Not all Indian states have a systemic understanding of disease burden distribution and time trends. In this context, we examined the relationship between disease burden (in DALYs and DALYs per hundred thousand population) and state health budget allocations for 5 years from 2015 to 2019. Since the COVID-19 pandemic threw healthcare allocation and spending patterns of their normal trajectory, we included a more recent pre-pandemic five-year period. For additional context and insight, we also looked at the Human Development Index (HDI) value for Indian states in the same period. This evidence can also help increase awareness and forewarning about the need for increased healthcare spending in India.

## Methods

We retrieved health budget allocation data from each of the Indian states and central data repositories (10, 11). The disease burden (in DALYs) was obtained from country-level data for India from the Global Burden of Diseases, Injuries, and Risk Factors Study (12) by the Institute for Health Metrics and Evaluation's Global Burden of Disease Studies (13). HDI values were extracted from available data sources for each Indian state and union territory (14). We examined the changes in disease burden as total DALYs and DALYs per hundred thousand of the population and changes in health budget allocation from 2015–2019. We explored the relationship between disease burden as total DALYs and DALYs per hundred thousand of the population with health budget allocation for each state government during the 5 years from 2015 to 2019 to understand the influence of population density. We also calculated percent changes for DALY and budget allocation across each year from 2015 to 2019 and an aggregate change for the 5-year period to visualize any trends in DALY changes and budget allocation among the states. Pearson's product-moment correlation coefficient statistic and graphical representation were used to observe the state-wise trends. *P*-value < 0.05 (*p* < 0.05) considered as significant. To understand the allocative efficiency, we further explored the budget allocation in Indian rupees per DALY for all the states from 2015 to 2019.

HDI measures the overall achievement in the health, education, and income aspects. For an external validation of the disease burden data, the standard of living as per HDI was explored to give more insight into the results. The correlation between state-wise total DALY and HDI were analyzed to understand the relationship between the overall development and the corresponding change in disease burden and for insights into the observed state-level trends. We also explored the total disease burden as total DALYs and DALYs per hundred thousand estimated under the broad category as non-communicable (NCDs), communicable (CDs), and injuries/accidents to the state health budget allocation over 5 years. We used Microsoft Excel (15) for data visualization and STATA software version 16, for statistical analysis. For currency conversion, we used the 2019 yearly average exchange rate (1US$ = 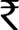 70.40) (16).

### Patient and Public Involvement Statement

All data used for the analysis were from secondary sources that are publicly available. No patients were involved in this study.

## Results

### A Transition in Disease Burden Over the Quinquennial Period

The disease burden (in DALYs) increased proportionally over the years 2015–2019 (Supplementary Figure 1). Jharkhand, Jammu and Kashmir, Bihar, Punjab, Tripura, Madhya Pradesh (MP), Uttar Pradesh (UP), and Chhattisgarh states had the greatest variation in disease burden over 5 years (Appendix I). Other states showed minor changes over time. The states where the change in DALYs over 5 years is low suggest that the increase in burden is not exceedingly high and may well be under control.

### Realignments in Health Budget Allocation Over the Quinquennial Period

From 2015 to 2019, states such as Assam, Arunachal Pradesh (AP), and MP saw more than 150 percent increases in health budget allocation, whereas Tripura saw only a 9% increase. In 2016, Tripura and Haryana received less budget allocation than in 2015, and in 2017, Punjab, Nagaland, and Manipur received less allocation than in 2015. Delhi's share of the total budget increase was higher than Meghalaya's (Supplementary Figure 2). From 2015 to 2019, most states' total budgets showed an increase in health budget allocation, except for Tripura, Haryana, Jammu & Kashmir, Manipur, Punjab, and Telangana, where the share of the health budget in the total budget decreased.

### Disease Burden (DALYs) as a Health Budget Metric

**Figure 1:**
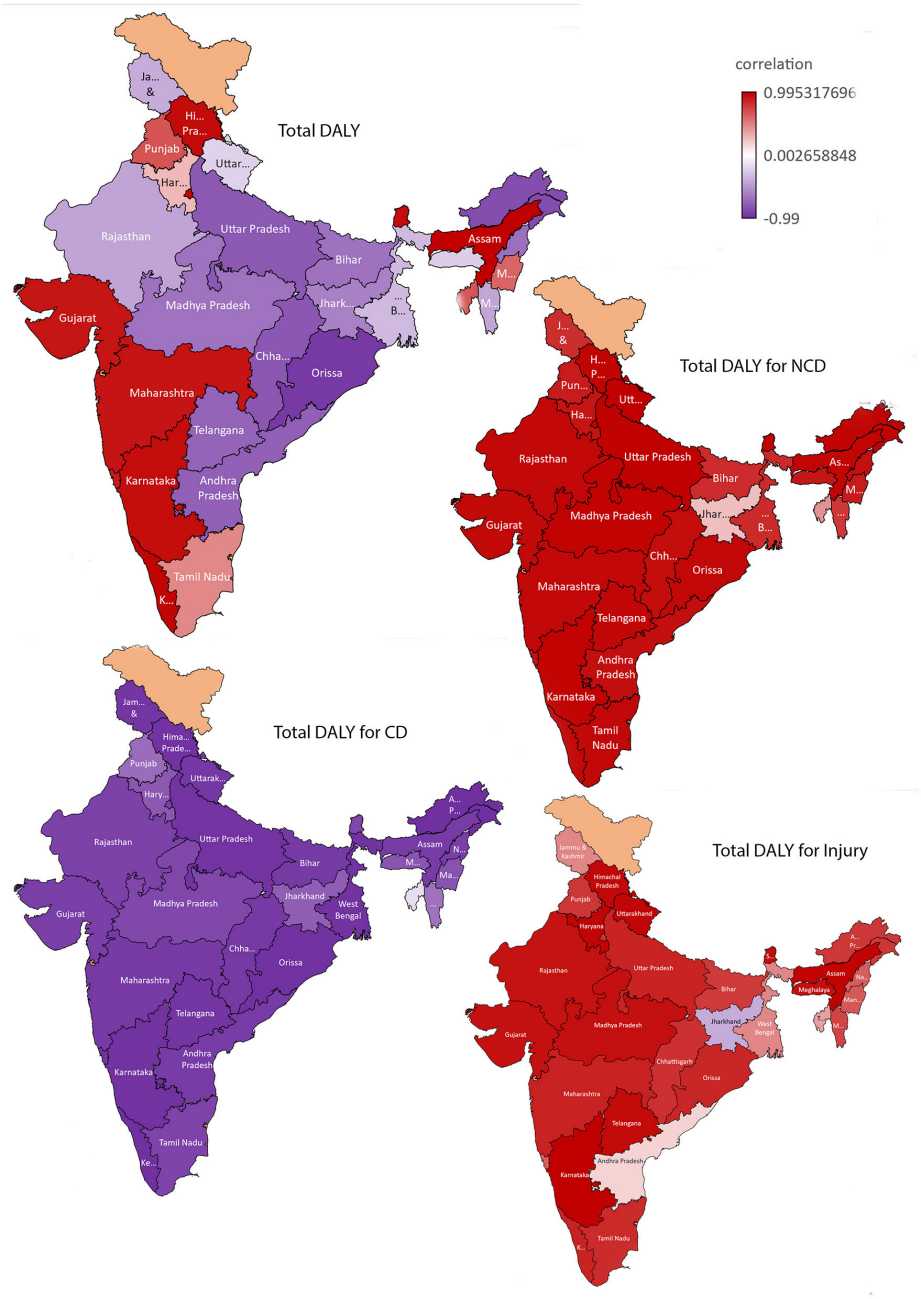
Correlation between budget allocation and disease burden [(estimated as Disability Adjusted Life Years (DALY)] in India: for Total, Non-Communicable diseases (NCD), Communicable diseases (CD) and injuries.

In most states, budget allocation is inversely proportional to DALYs. While the estimated DALYs vary unequally with an increase in health expenditure, there is a positive correlation between budget allocation and disease burden in some states in the Northern and North-eastern regions and along with the coastal Southwest India. However, only for the following states Assam, Delhi, Goa, Gujarat, Himachal Pradesh, Karnataka, Kerala, Maharashtra, Odisha, and Sikkim are statistically significant (*p* < 0.05) (Figure 1; Supplementary Table 1; Appendix II). There is a strong positive correlation between NCDs' disease burden and budget allocation for all Indian states except Jharkhand and Tripura with a weak positive correlation. Though, all the states except Bihar, Jammu & Kashmir, Jharkhand, Mizoram, Tripura, and West Bengal are statistically significant (*p* < 0.05) (Figure 1; Supplementary Table 1; Appendix III). In all Indian states except Tripura (weak negative correlation), there is a strong negative correlation between CDs disease burden and budget allocation. Even if, all the states except Haryana, Jharkhand, Madhya Pradesh, Manipur, Meghalaya, Mizoram, Punjab, and Tripura are statistically significant (*p* < 0.05) (Figure 1; Supplementary Table 1; Appendix IV).

There is a strong positive correlation between disease burden due to injuries/accidents (self-harm) and budget allocation for all Indian states except Jharkhand (weak negative correlation). Tripura, West Bengal, and J & K have moderate positive correlations. However, only for the following states Assam, Gujarat, Haryana, Himachal Pradesh, Karnataka, Madhya Pradesh, Meghalaya, Rajasthan, Sikkim, Telangana, and Uttarakhand are statistically significant (*p* < 0.05) (Figure 1; Supplementary Table 1; Appendix V).

### Disease Burden (DALYs per Hundred Thousand Population) as a Health Budget Metric

**Figure 2:**
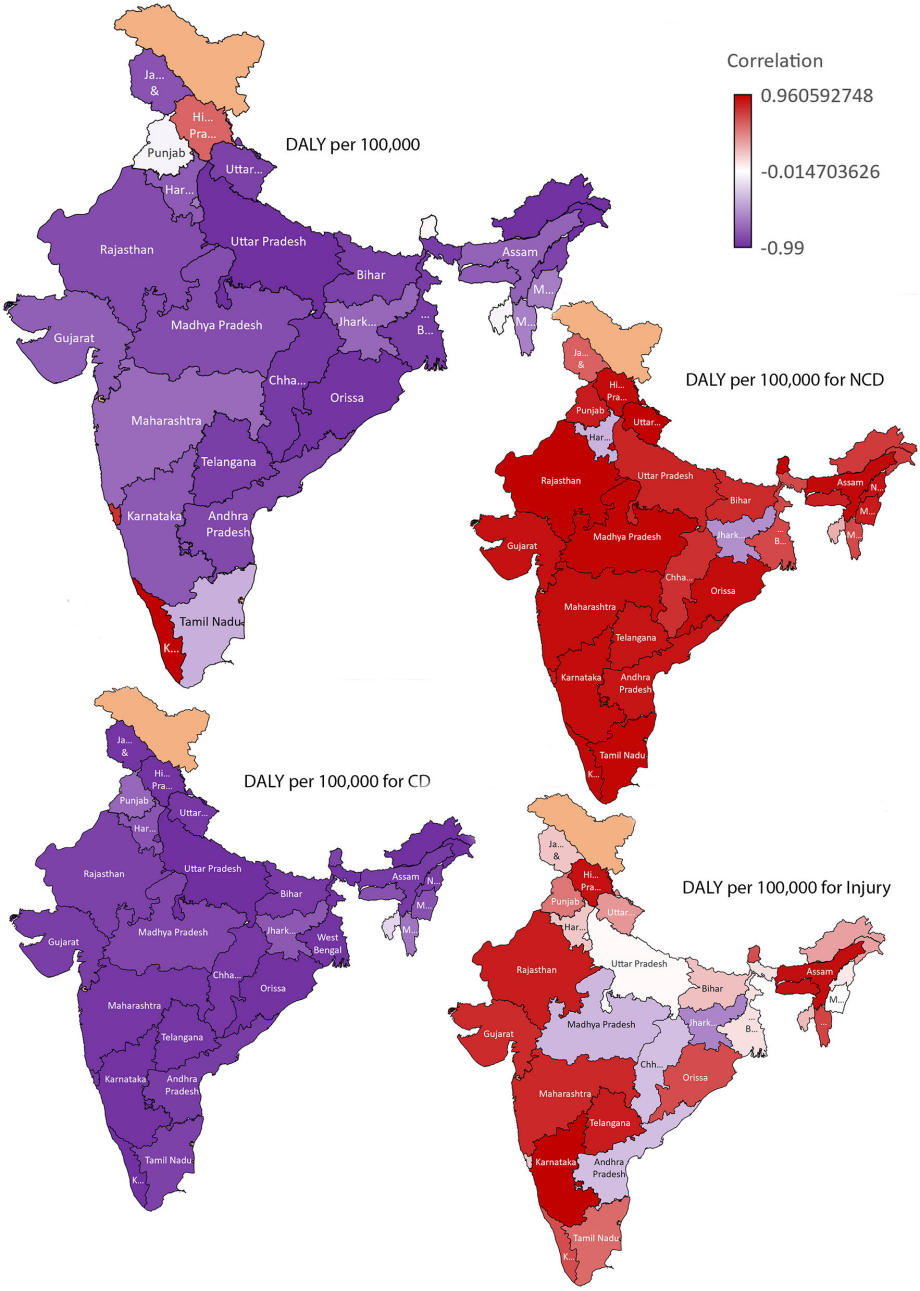
Correlation between budget allocation and disease burden (estimated as DALY) per 100,000 population in India: for Total, Non-Communicable diseases (NCD), Communicable diseases (CD) and injuries.

In most states, healthcare budget allocation is inversely proportional to DALYs per hundred thousand population. The correlation graph shows a decrease in DALYs per hundred thousand population with increased budget allocation. While the estimated DALYs per hundred thousand population vary unequally with an increase in health expenditure, there is a negative correlation between budget allocation and DALYs per hundred thousand population in most states except Kerala, Goa, and Himachal Pradesh (see Figure 2). There is a strong positive correlation between NCDs disease burden and budget allocation for all Indian states except Jharkhand and Haryana (moderate negative correlation) (Figure 2). In all Indian states, healthcare budget allocation is negatively correlated with DALYs per hundred thousand population in the case of CDs. DALY per hundred thousand population due to injuries and accidents shows a positive correlation with budget allocation, except in Jharkhand, MP, Andhra Pradesh, and Chhattisgarh, where there is a moderate negative correlation (Figure 2).

### State Budget Allocation per DALY for Overall Disease Burden

The estimated budget allocations per DALY for each state assess the efficiency of healthcare budget spending. A decrease in total DALY and an increase in budget allocation offer a higher allocated budget per DALY. Goa, Delhi, and most northeast states except Assam have higher budget allocations per DALY (Supplementary Figure 3). Goa had the highest budget allocation per DALY for the 5 years, with an estimated 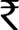
34,260 (US $ 486·66) in 2019. Bihar remained the state with the lowest budget allocation per DALY for the 5 years, with an estimated 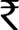 2,408 (US $ 34.20) in 2019 (Supplementary Figure 4).

### DALY and the Human Development Index

The trend in DALY and HDI values among Indian states from 2015 to 2019 was plotted (Supplementary Figure 5). Uttar Pradesh, Bihar, Madhya Pradesh, Jharkhand, and Rajasthan have lower HDI values than other states. Contrary to the previous trend, Goa, Himachal Pradesh, Punjab, and Sikkim had a low disease burden but a high HDI over time. However, both DALY and HDI values increased over the period in Karnataka and Maharashtra. In contrast, DALY and HDI values decreased over the period in Nagaland, Telangana, and Tripura.

## Discussion

While health statuses are improving, there are greater differences in disease profiles between states in India. There are major inequalities between states, and the states are at different levels of epidemiological transition (17). There were striking differences between the states in the level of burden from NCDs, with an overall increase in the burden of NCDs among all states. Although the burden of infectious diseases is declining, the burden of injuries varies by states. From 2015 to 2019, most of the states' total budgets showed an increase in health budget allocation, but the disease burden also increased proportionally over the same period. For most of the states the healthcare budget allocation had an inverse relationship with communicable diseases and an identical linear relationship with non-communicable diseases. The positive correlation, along with the strength of the relationship between budget allocation and DALYs, suggests the significance of increased budget allocation and its impact on reducing disease burden, especially CD burden. However, even with increased health budgets, the increasing NCD burden could be multifactorial including chronicity of illness, lifestyle changes, better disease detection. Etc and require further deliberations in understanding the reasons for these observed trends. Further, to be mindful of the inequalities between states while interpreting the trends in disease burden across the years and with varying budget allocations.

It is worth noting that there were significant differences in allocated budget per DALY due to differences in disease burden levels between neighboring states with relatively higher levels of development indicators. Though, the neighboring states are at a similar and more advanced epidemiological transition stage. The North-East Indian states have the highest budget allocation per DALY. North-East states are smaller, less populous states and are at a lower-middle stage of epidemiological transition with different disease burdens. The states with the lowest budget allocation per DALY include Empowered Action Group states (18). Empowered Action Group include eight states, Bihar, Jharkhand, MP, Chhattisgarh, Orissa, Rajasthan, Uttar Pradesh and Uttarakhand which are geographically larger, highly populous and have a relatively lower development indicator. These states are at a similar and less advanced epidemiological transition stage and were also left behind in the demographic transition. Thus, the study observation indicates that state population size significantly affects the budget allocation per DALY ratio, as larger states have a higher total DALY per capita.

With an increased budget allocation, there was a noticeable reduction in the disease burden due to CDs. CDs' occurrence can often be predicted to the maximum extent, and precautions can be taken in advance. The CD prevention and treatment programmes worked regardless of geographic or seasonal differences. So, the budget allocation may have been well followed by the origin of the CDs at various time points and geographical locations. In this way, budget allocations can often be predicted and planned. It is evident from the results that government spending on the health sector is increasing, but it is inadequate to cover the increasing disease burden of NCDs. In addition, the allocated healthcare budget may have been utilized to control the severity of these diseases by expanding treatment coverage and healthcare facilities. This raises questions about future budgeting and healthcare spending on NCD prevention. More focus must be provided to NCDs since the current spending does not help curtail NCDs' disease burden. Early interventions, timely measures, and investing in public health infrastructure and resources can reduce the disease burden (19).

Globally, there is no clear trend of relationship between increase in healthcare spending and health needs (20). In India, usually there is a preference for a greater spending on curative health. However, a health system with higher public spending on overall health tends to improve financial protection for individuals (20). A study which looked at the non-communicable diseases and injuries (NCDI) expenditure from the Indian Union budget for 2012–13 to 2016–17 reported total spending on NCDI by the government is low at <0.5% of GDP, which is little more than one-fourth of the total health spending of the country. The gap between spending and DALYs is the greatest for the economically vulnerable states. Also, the states with high poverty levels also have low per capita expenditure on NCDI (21). The research and development (R&D) budget of the Ministry of Health and Welfare in 2018 compared with the results of the 2015 Korean National Burden of Disease showed that R&D allocations were not focused on minimizing the burden of disease in terms of economic burden and DALYs in Korea (22). Another study on the Korean healthcare system found that the budget provided was insufficient to address the mental and behavioral disorders disease burden (23).

Unspent funds, under-allocation, and poor allocation choices in the healthcare sector due to fragmented governance across different states in India may also reduce allocative efficiency. This failure to spend is not unique to any state or health program, and many vertical health programmes have suffered from the same malaise (24). The Financial Commission's recommendations and fiscal devolution restrict the central government with fewer resources at its disposal. Hence, to increase the center's direct investment in social sectors, the states must prioritize their health sector. The government's disease prevention plans already in place must be reviewed and revised. Although the observed trends between the association of disease burden and the HDI may not be completely independent of one another, plausible reasons which question the relationship needs to be explored. The observed trends show that the budget allocation must be modified based on the disease burden of each state. A data-driven health planning and budget allocation specific to each state might reduce the increasing disease burden in the respective states. We felt that the proportion of the state budgets was not associated with the size of the problem; health policies and programs were not associated with the proportion of the health budget allocated to health. We feel that the economic burden of diseases should be one of the core principles that need careful consideration in deciding health budget allocation in India. Also, more research is needed on allocation principles for non-disease-specific categories. State-level health policies must transform to adapt to chronic and diverse disease patterns. At the same time, the increase in domestic exposure to foreign infectious diseases due to increased international travel emphasizes the importance of having well-developed, coordinated health systems. Rapid environmental changes are altering infectious disease patterns, and new infectious diseases are emerging in India. The COVID-19 pandemic recently reduced India's geographical and seasonal diversity and has brought attention to the healthcare sector and its interconnections. Lessons from the current health crisis are crucial. However, healthcare budget allocation must not be subject to “saliency bias,” where a recent event may be a six-sigma event that will not be repeated in the future (25).

### Limitation

This study used annual state health budget allocation data instead of actual health expenditure. The federal allocation for health care varies by state budget system and is not included in our analysis. So, depending on the central share, the actual budgeted healthcare expenditure may vary slightly. Similarly, the components of total health expenditure and its state level variation in DALY was omitted due to lack of specific data. Full details, including data limitations, of the GBD study, have been published elsewhere (12, 26). Other limitations for India include incorrect cause of death reporting and a difference in state-by-state coverage. There may be data gaps in the estimation process of health information systems across India. Objective outcomes focusing on disease burden estimates may suffer from the discernible that they might include only what the government wants to monitor. Also, in India, most health spending is done “out of pocket” (27, 28). Our analysis did not include out of pocket as a percentage of total health expenditure. Our focus was on the GBD project's three disease categories. We looked at the budget distribution in relation to DALYs, but not the economic burden of disease.

### Way Forward

We observed a remarkable variation in disease burden across states. However, the health budget allocation by each state was not based on their disease burden. The variation in disease burden across India indicates the existence of differences in underlying social, behavioral, or biological and other unknown risk factors. The meteoric rise in the burden of NCDs in all states due to this combination of risks indicates unequivocally that major efforts need to be put in place to control their impact in every state before the situation gets out of control. This variability in trends among NCDs, CDs, and Injuries indicates that policy and health system interventions to tackle their increasing burden must be informed by the specific trends, and effective action to improve health must finally be based on the specific health situation of each state. This point is elucidated by significant variations in the burden and health budget allocation in states with physical proximity and is at similar development and epidemiological transition levels. It would be helpful to have a cost per QALY or cost per DALY database for India. Budgets for health care are inadequate to curtail the disease burden in many Indian states, and the allocation system should be improved. Also, the allocated money may not be working as intended due to unidentified losses. The disparity between government spending and disease burden must be investigated. The current paradigm will continue to distort and bias service delivery until health systems adopt an evidence-based cost-effectiveness approach. DALY per hundred thousand and allocated budget per DALY may be more appropriate measures, and decentralized and improved burden estimates would complement states' specific budget allocation for health in the future.

## Data Availability Statement

The original contributions presented in the study are included in the article/Supplementary Material, further inquiries can be directed to the corresponding author.

## Author Contributions

BB: conceptualization, data curation, formal analysis, investigation, methodology, inputs on original draft, and review and editing. SK and AS: data curation, formal analysis, and original draft. All authors contributed to the article and approved the submitted version.

## Funding

The Department of Health Research, Government of India Funds the Health Technology Assessment Resource Centre, ICMR-NIE. Funders had no role in the conceptualization, conduction, and manuscript preparation.

## Conflict of Interest

The authors declare that the research was conducted in the absence of any commercial or financial relationships that could be construed as a potential conflict of interest.

## Publisher's Note

All claims expressed in this article are solely those of the authors and do not necessarily represent those of their affiliated organizations, or those of the publisher, the editors and the reviewers. Any product that may be evaluated in this article, or claim that may be made by its manufacturer, is not guaranteed or endorsed by the publisher.
