# Supplementary material for: Is State-Wise Healthcare Budget Allocation Consistent With the Disease Burden in India? A Quinquennial Account (2015–2019)
Source: Front Public Health. 2022 Jun 28;10:893257. doi: 10.3389/fpubh.2022.893257 (PMC9273767; doi:10.3389/fpubh.2022.893257)
Supplement: Supplementary file 1 [file Data_Sheet_1.PDF]

Online Supplementary Material

Of manuscript:
Bagepally B.S., Sajith Kumar S., Sasidharan A. "Is state-wise healthcare budget allocation consistent with the disease burden in India? a quinquennial account (2015–2019)"

1. **Supplementary Figures - Page 2 to 6**
2. **Supplementary Table - Page 7**
3. **Appendix - Page 8 to 30**

Supplementary Figure 1. Change in disease burden in India during 2015-19 period

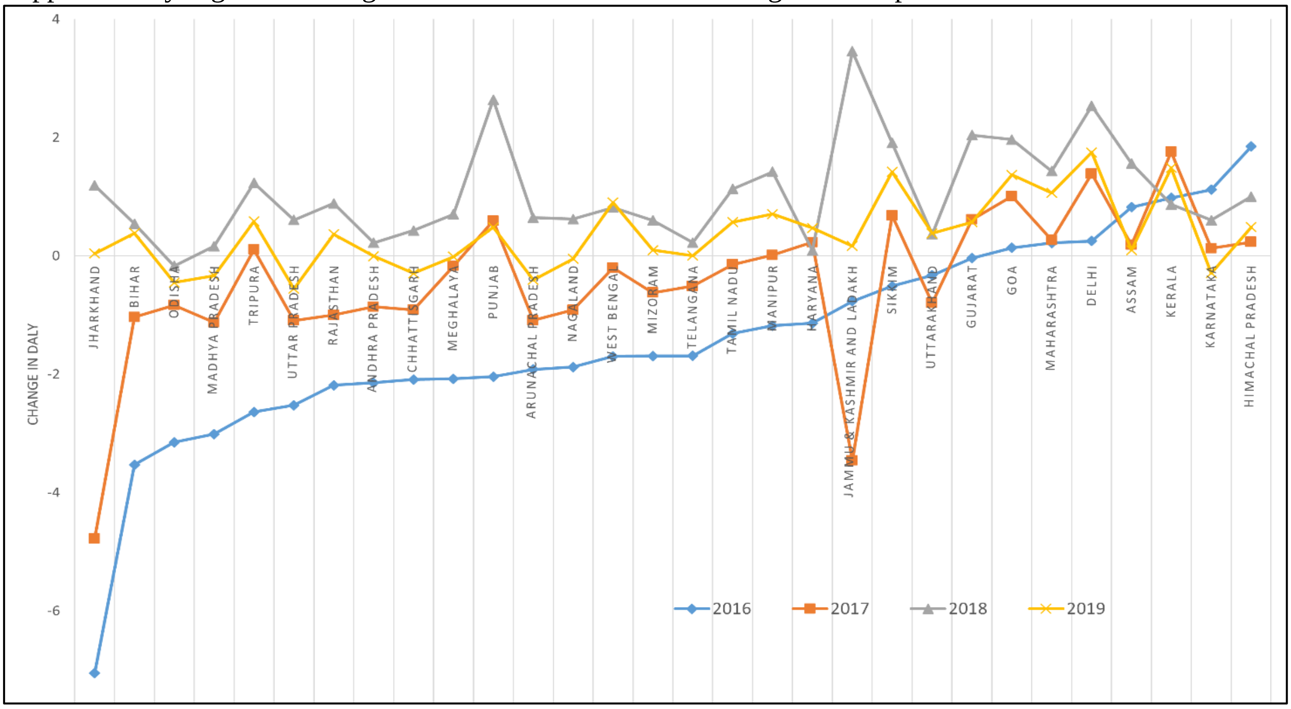

[Line graph with the Y-axis represents change in DALY across years from 2015 to 2019 for individual states in India]

Supplementary Figure 2. Change in health budget allocation in India during 2015-2019

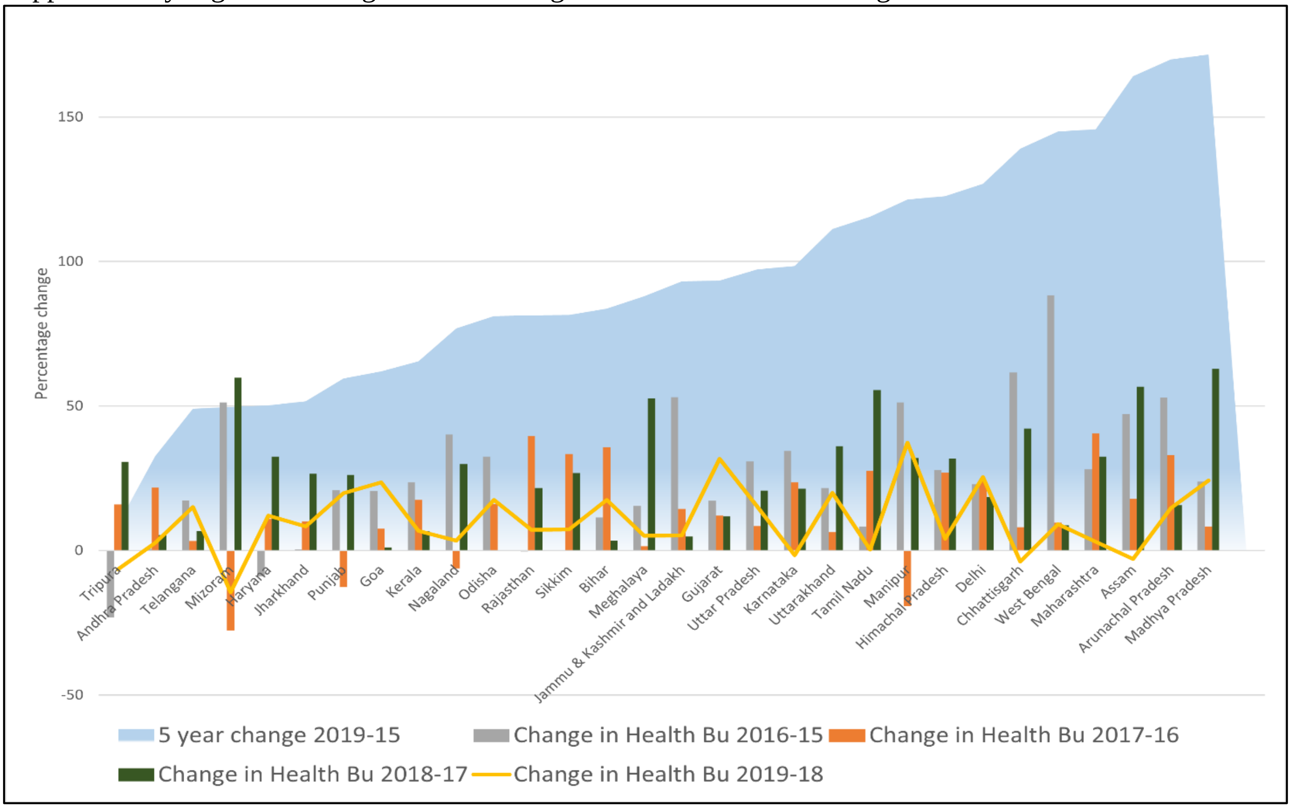

[Area chart with the Y-axis represents a five-year percentage change in Budget allocation (from 2015 to 2019) for individual states in India. Clustered column and line graph represents annual change in budget allocation for individual states]

Supplementary Figure 3. Change in Budget allocation per DALY in India during 2015-19 period

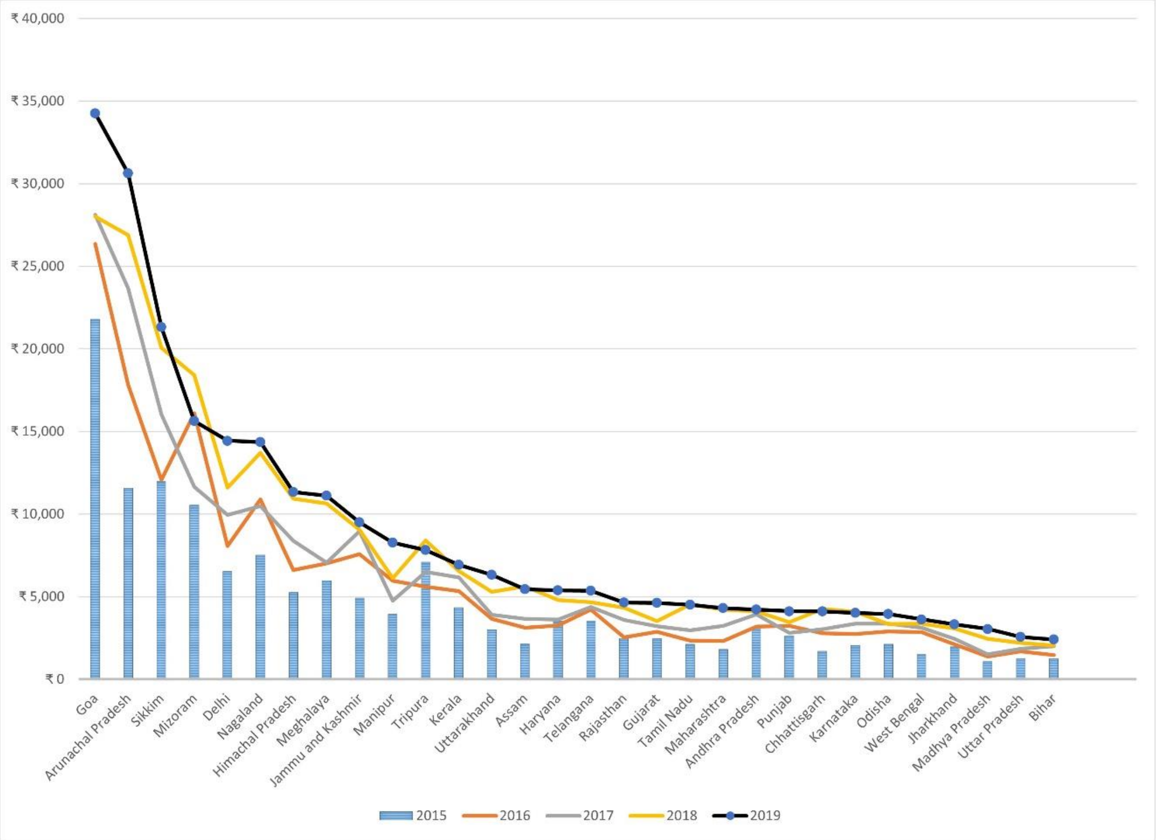

[Line graph and clustered column chart with the Y-axis represents change in budget allocation per DALY for five years from 2015 to 2019 for individual states in India]

Supplementary Figure 4. Tree map showing the Budget allocation per DALY in Indian states during 2019

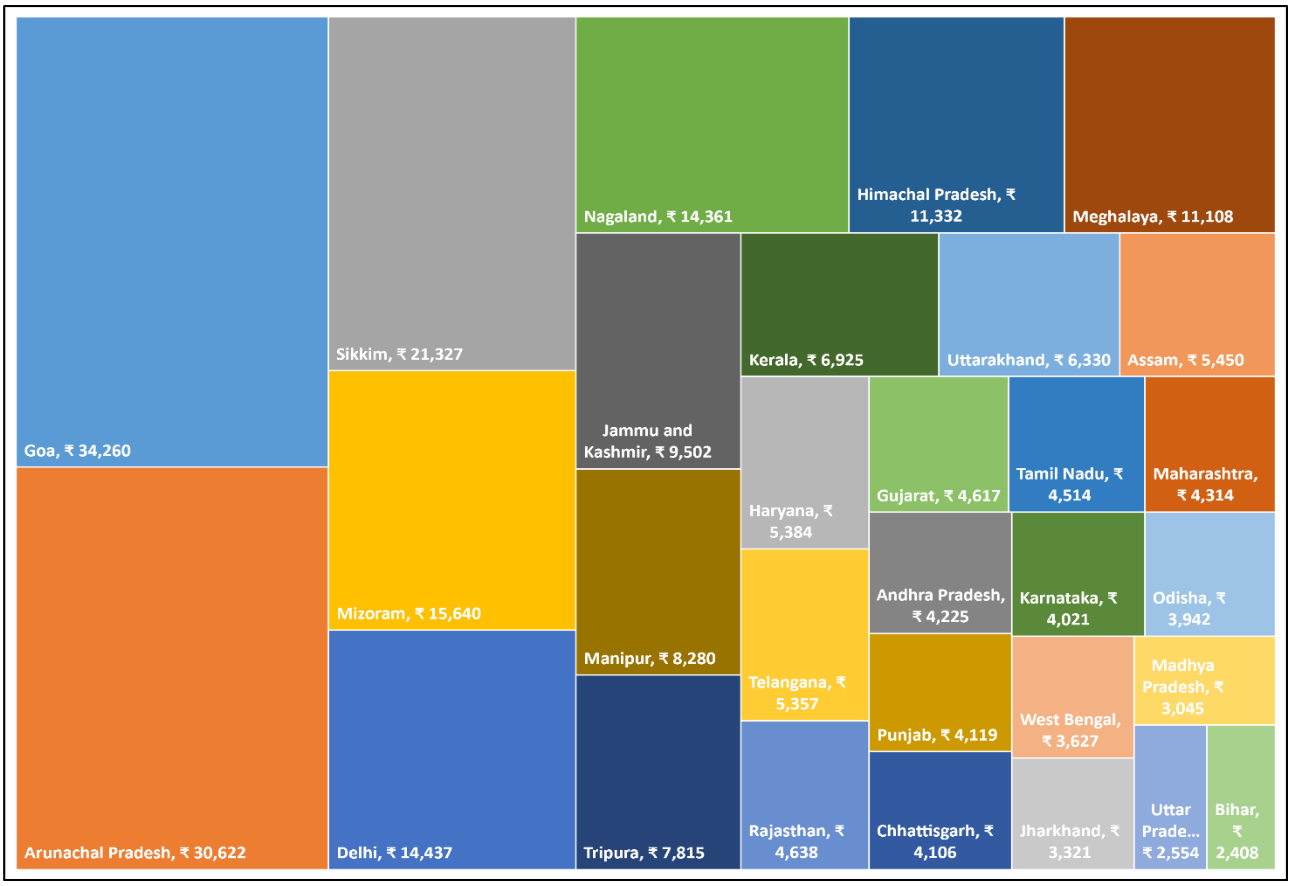

Supplementary Figure 5. Trends in total Disease burden and HDI in India

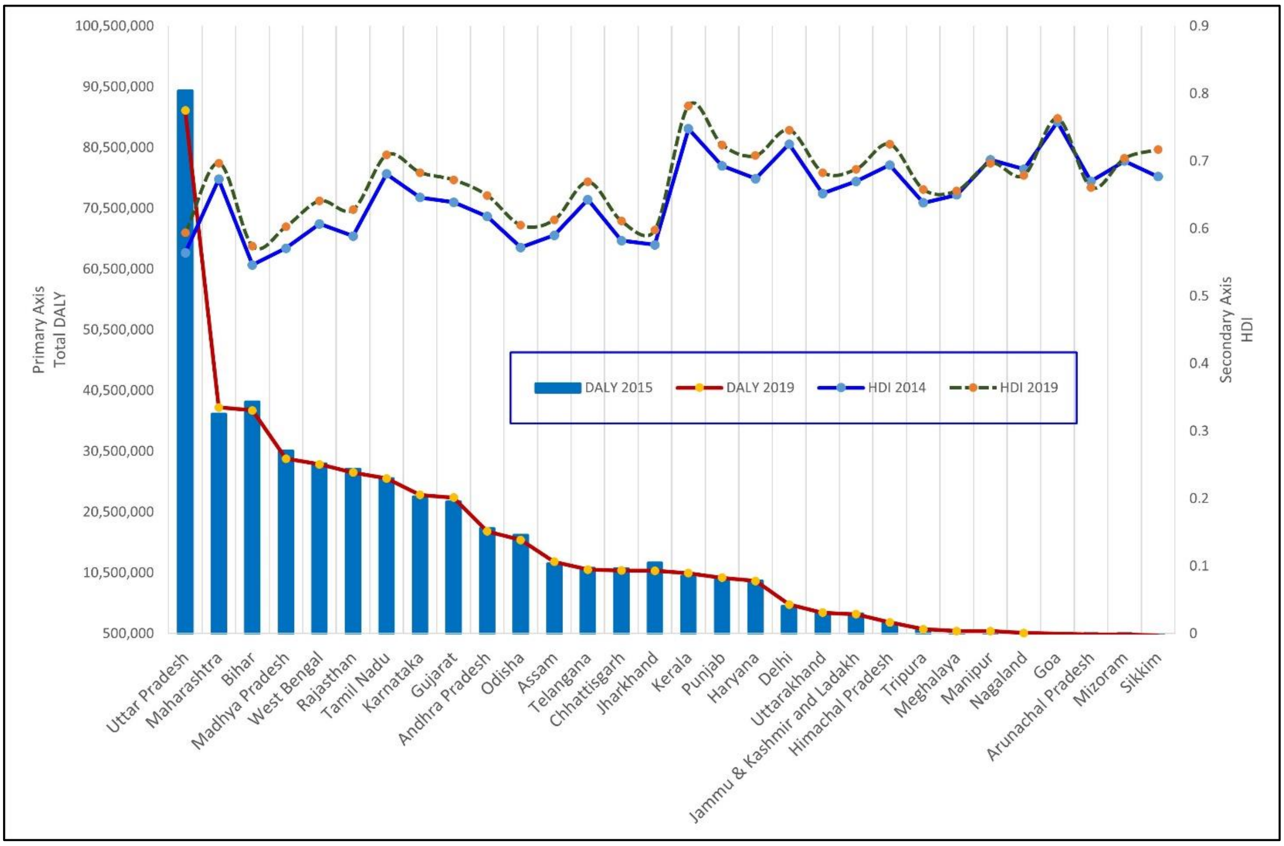

[Clustered column chart and line graph with the primary Y-axis represents total DALY for individual states in India during 2015 and 2019. Line graph with the secondary Y-axis represents Human Development Index (HDI) values for individual states in India during the same period.]

Supplementary Table 1. Correlation between budget allocation and disease burden [(estimated as Disability Adjusted Life Years (DALY)] in India: for Total, Non-Communicable diseases (NCD), Communicable diseases (CD) and injuries.

State	**Total**		**CD**		**NCD**		**Injury**	
	correlation	p value	Correlation	p value	Correlation	p value	Correlation	p value
Andhra Pradesh	-0.758	0.138	-0.933	0.021**	0.932	0.021**	0.171	0.784
Arunachal Pradesh	-0.851	0.067	-0.991	0.001**	0.973	0.005**	0.774	0.124
Assam	0.995	0.00**	-0.933	0.021**	0.966	0.007**	0.975	0.005**
Bihar	-0.671	0.215	-0.940	0.017**	0.819	0.090	0.764	0.133
Chhattisgarh	-0.805	0.100	-0.950	0.013**	0.942	0.017**	0.802	0.102
Delhi	0.981	0.003**	-0.917	0.028**	0.991	0.001**	0.786	0.115
Goa	0.896	0.040**	-0.966	0.008**	0.929	0.023**	0.618	0.267
Gujarat	0.914	0.030**	-0.919	0.027**	0.952	0.013**	0.924	0.025**
Haryana	0.276	0.653	-0.800	0.104	0.909	0.032**	0.970	0.006**
Himachal Pradesh	0.952	0.013**	-0.975	0.005**	0.970	0.006**	0.958	0.010**
Jammu & Kashmir and Ladakh	-0.393	0.512	-0.964	0.008**	0.812	0.095	0.468	0.426
Jharkhand	-0.603	0.281	-0.770	0.128	0.249	0.686	-0.398	0.507
Karnataka	0.945	0.015**	-0.977	0.004**	0.980	0.003**	0.992	0.001**
Kerala	0.977	0.004**	-0.988	0.002**	0.973	0.005**	0.852	0.067
Madhya Pradesh	-0.667	0.219	-0.876	0.051	0.980	0.003**	0.929	0.023**
Maharashtra	0.917	0.028**	-0.939	0.018**	0.957	0.011**	0.860	0.062
Manipur	0.603	0.282	-0.825	0.085	0.888	0.044**	0.650	0.235
Meghalaya	-0.239	0.699	-0.814	0.093	0.903	0.036**	0.931	0.022**
Mizoram	-0.422	0.479	-0.669	0.217	0.772	0.126	0.728	0.163
Nagaland	-0.682	0.205	-0.921	0.026**	0.930	0.022**	0.630	0.255
Odisha	-0.940	0.018**	-0.983	0.003**	0.962	0.009**	0.844	0.072
Punjab	0.670	0.216	-0.702	0.186	0.885	0.046**	0.777	0.122
Rajasthan	-0.434	0.465	-0.906	0.034**	0.957	0.010**	0.918	0.028**
Sikkim	0.944	0.016**	-0.885	0.046**	0.978	0.004**	0.937	0.019**
Tamil Nadu	0.465	0.430	-0.894	0.041**	0.959	0.010**	0.823	0.087
Telangana	-0.740	0.153	-0.962	0.009**	0.964	0.008**	0.948	0.014**
Tripura	0.661	0.224	-0.161	0.796	0.419	0.482	0.365	0.545
Uttar Pradesh	-0.793	0.109	-0.971	0.006**	0.984	0.002**	0.854	0.066
Uttarakhand	-0.212	0.733	-0.954	0.012**	0.990	0.001**	0.970	0.006**
West Bengal	-0.328	0.590	-0.977	0.004**	0.826	0.085	0.469	0.425

****** significant at 95% confidence level ($p<0.05$)

Appendix I. Change in disease burden among Indian states during 2015-19 period*

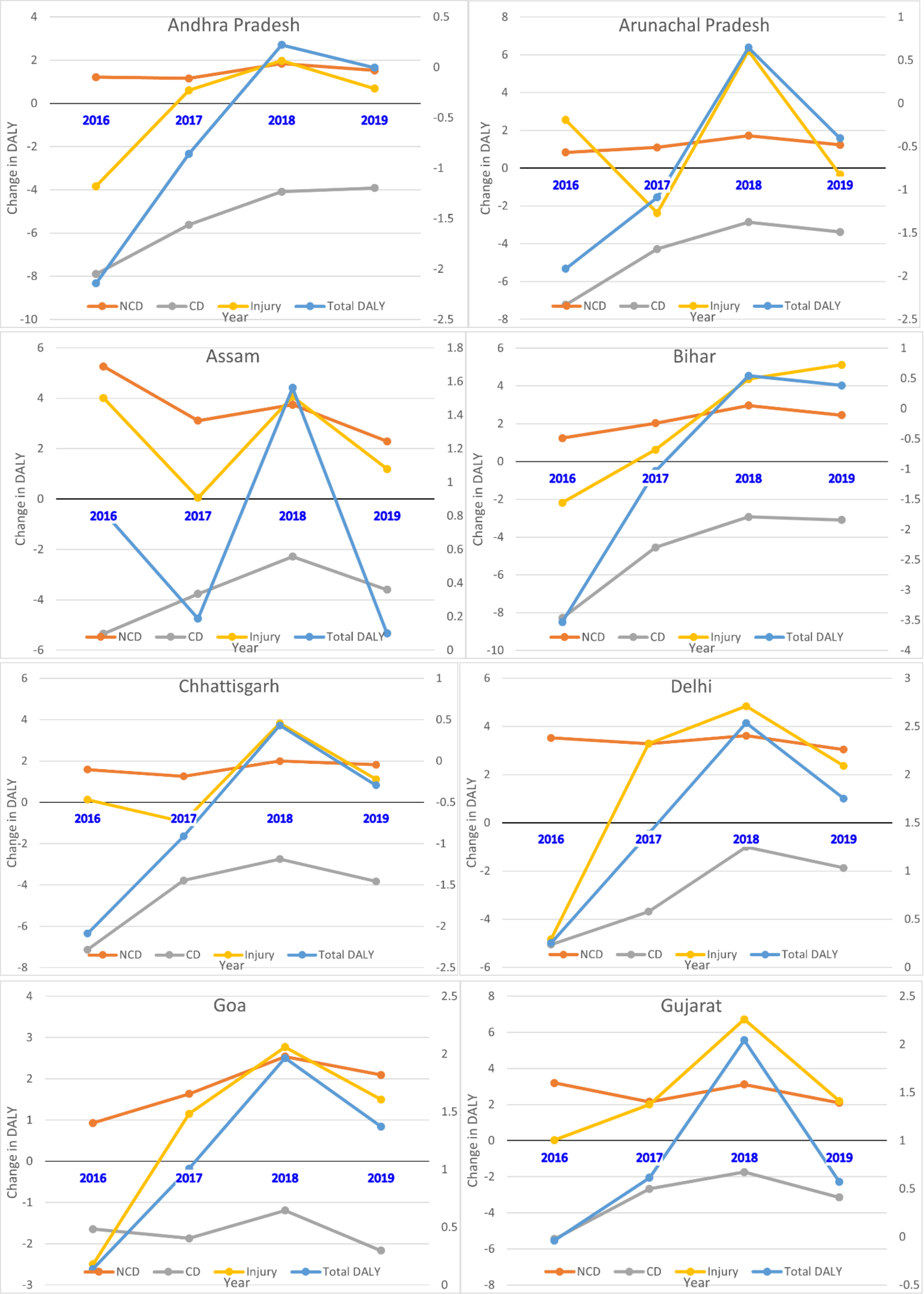

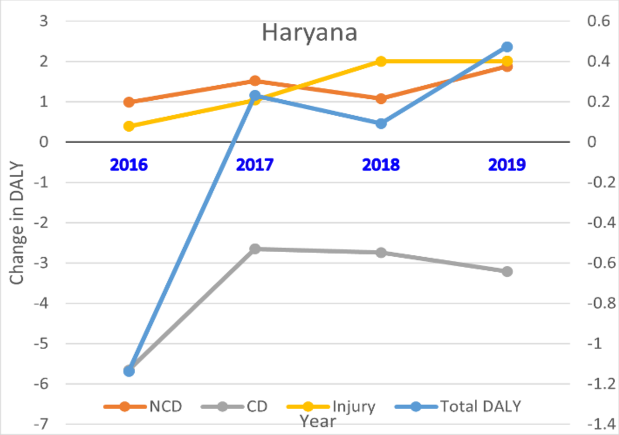
Haryana
Change in DALY
2016
2017
2018
2019
NCD
CD
Injury
Total DALY
Year

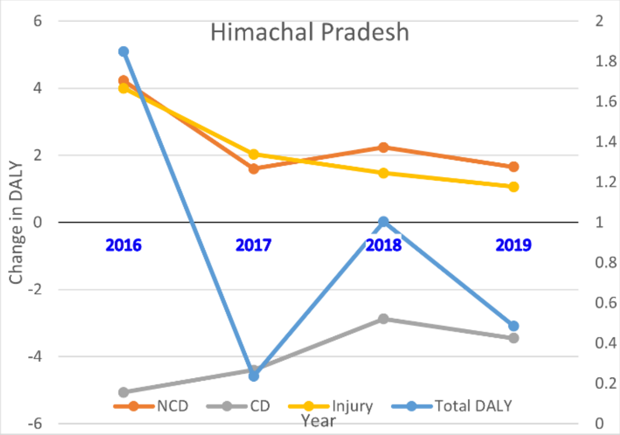
Himachal Pradesh
Change in DALY
2016
2017
2018
2019
NCD
CD
Injury
Total DALY
Year

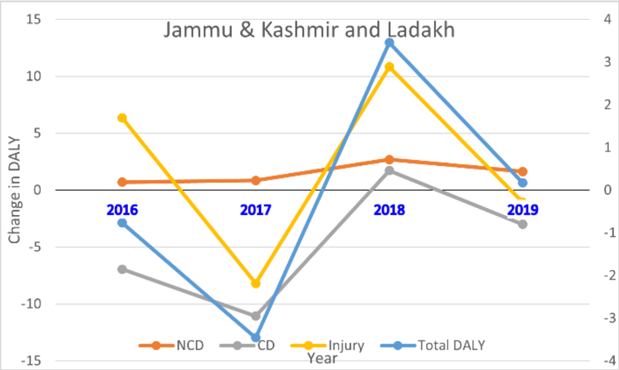
Jammu & Kashmir and Ladakh
Change in DALY
2016
2017
2018
2019
NCD
CD
Injury
Total DALY
Year

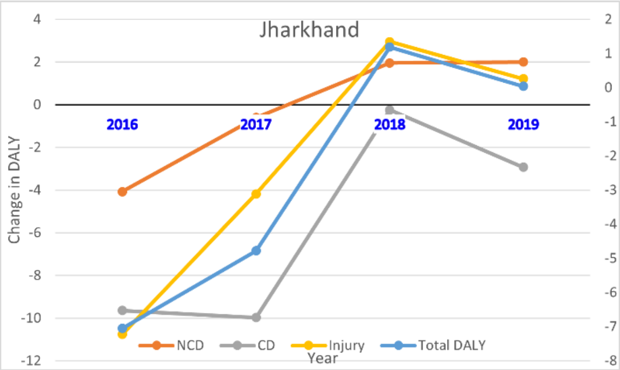
Jharkhand
Change in DALY
2016
2017
2018
2019
NCD
CD
Injury
Total DALY
Year

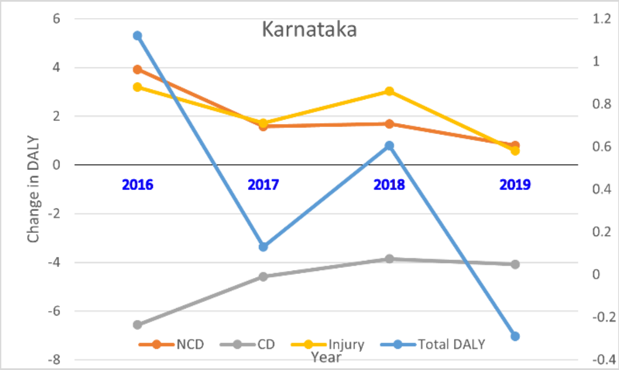
Karnataka
Change in DALY
2016
2017
2018
2019
NCD
CD
Injury
Total DALY
Year

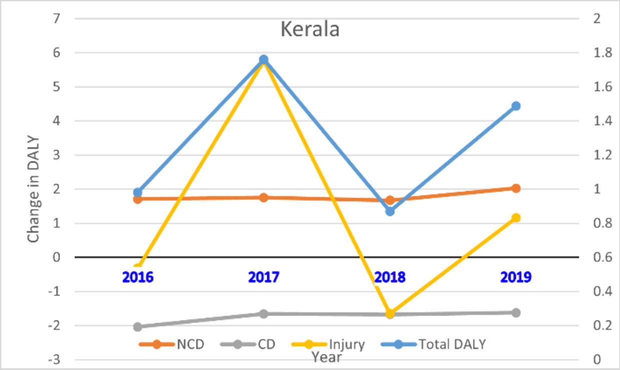
Kerala
Change in DALY
2016
2017
2018
2019
NCD
CD
Injury
Total DALY
Year

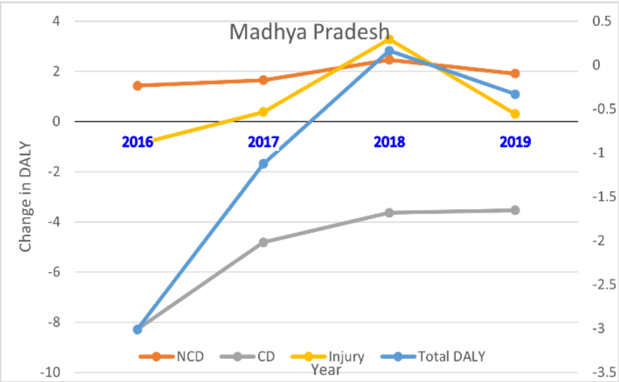
Madhya Pradesh
Change in DALY
2016
2017
2018
2019
NCD
CD
Injury
Total DALY
Year

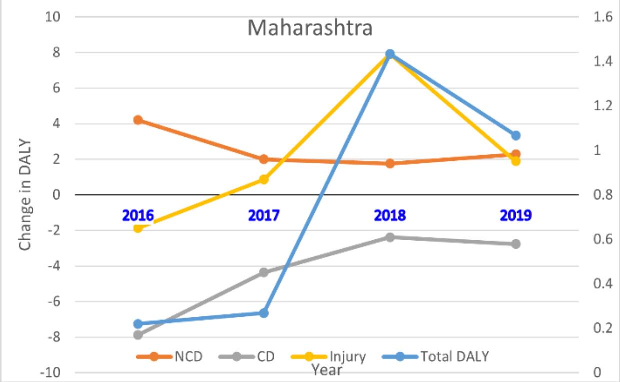
Maharashtra
Change in DALY
2016
2017
2018
2019
NCD
CD
Injury
Total DALY
Year

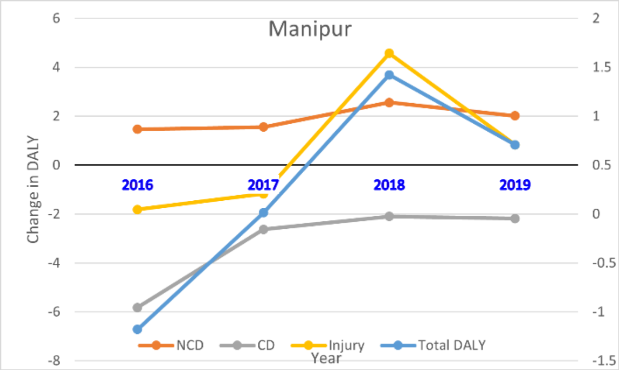
Manipur
Change in DALY
2016
2017
2018
2019
NCD
CD
Injury
Total DALY
Year

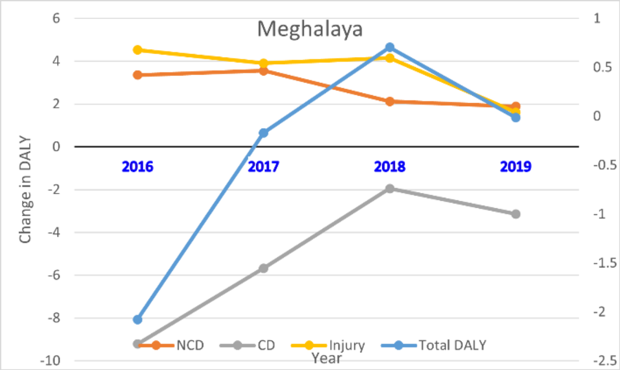
Meghalaya
Change in DALY
2016
2017
2018
2019
NCD
CD
Injury
Total DALY
Year

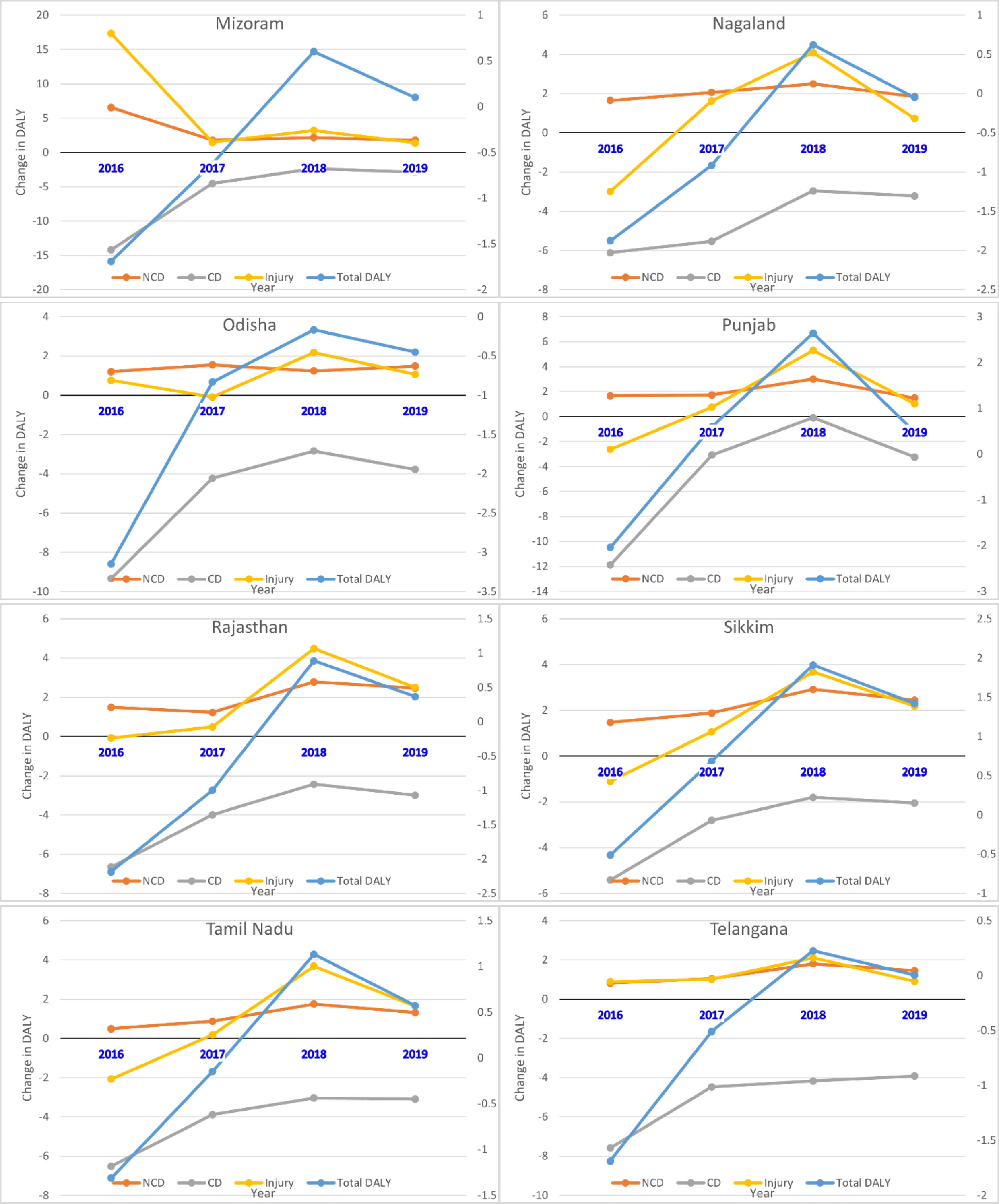
Mizoram
Nagaland
Odisha
Punjab
Rajasthan
Sikkim
Tamil Nadu
Telangana
Change in DALY
Year
2016
2017
2018
2019
NCD
CD
Injury
Total DALY

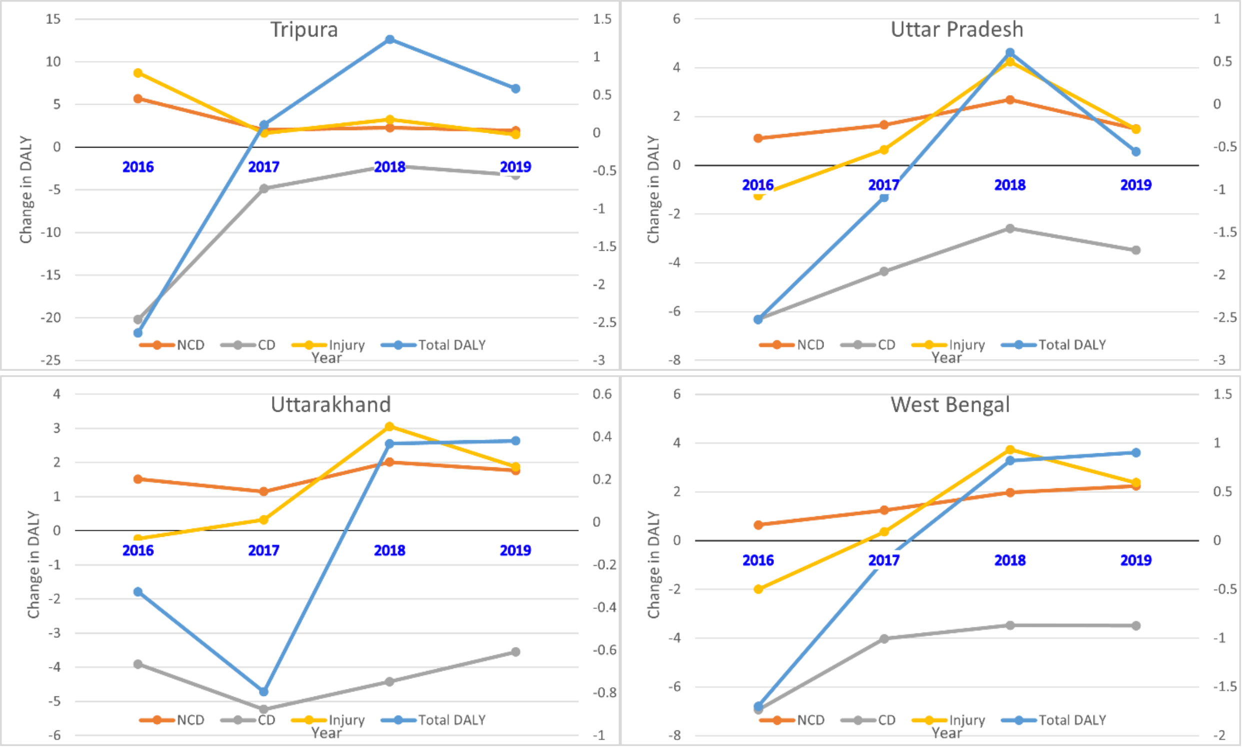

*[Line graph with the primary Y-axis represents annual change in DALY for NCD, CD and injury from 2016 to 2019 for individual states in India. Secondary Y-axis represents annual change in Total DALY from 2016 to 2019 for individual states in India]

Appendix II. Change in Disease burden and Health budget allocation among Indian states

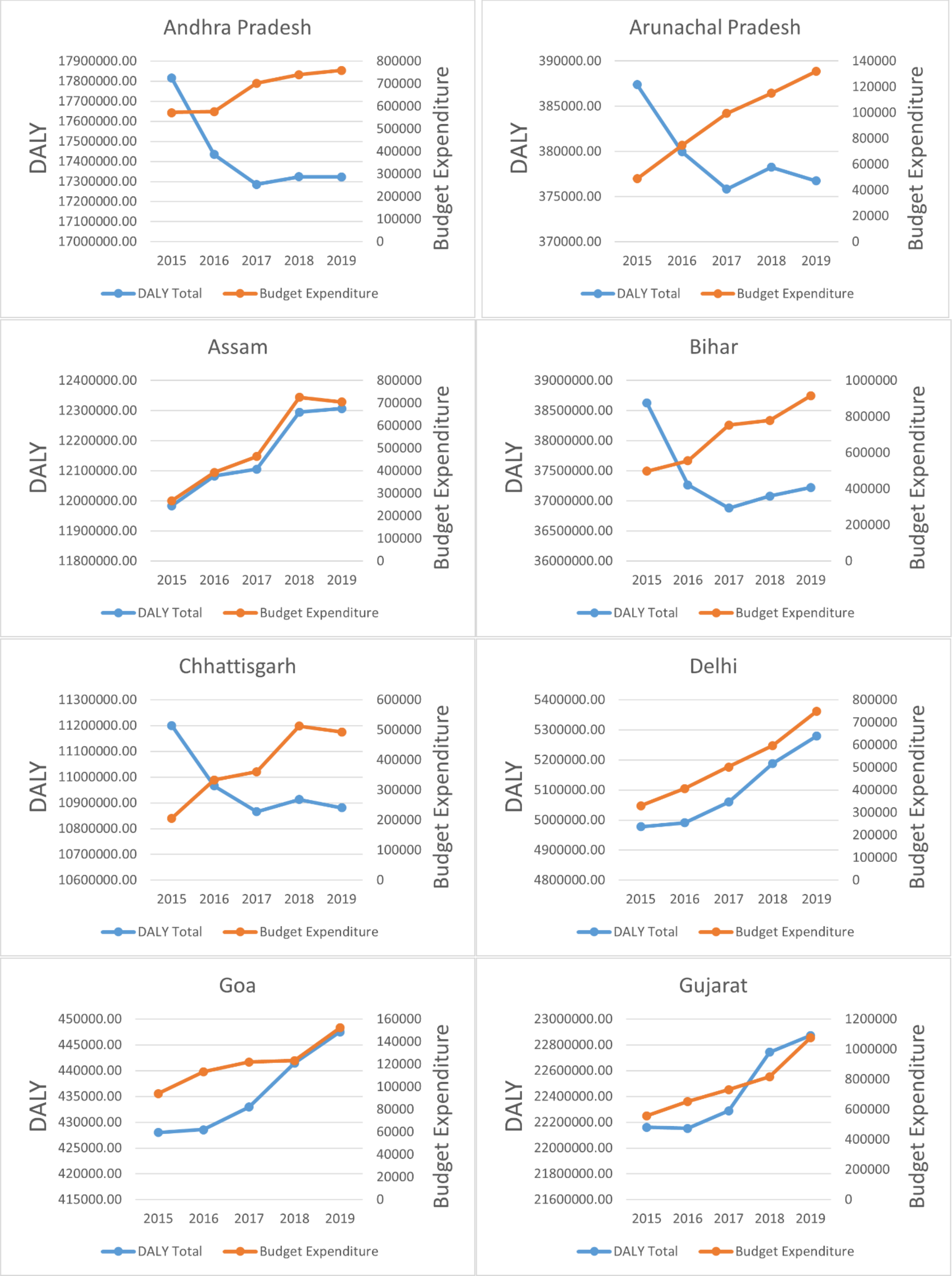

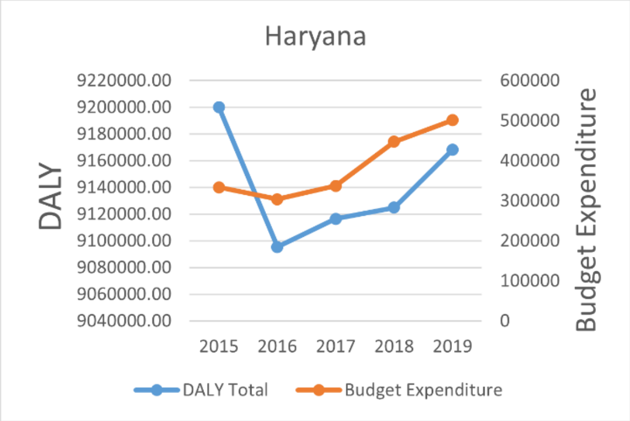
Haryana
DALY
9220000.00
9200000.00
9180000.00
9160000.00
9140000.00
9120000.00
9100000.00
9080000.00
9060000.00
9040000.00
600000
500000
400000
300000
200000
100000
0
Budget Expenditure
2015 2016 2017 2018 2019
DALY Total
Budget Expenditure

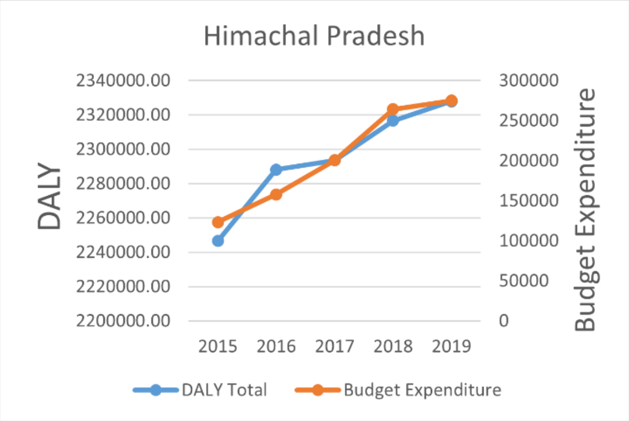
Himachal Pradesh
DALY
2340000.00
2320000.00
2300000.00
2280000.00
2260000.00
2240000.00
2220000.00
2200000.00
300000
250000
200000
150000
100000
50000
0
Budget Expenditure
2015 2016 2017 2018 2019
DALY Total
Budget Expenditure

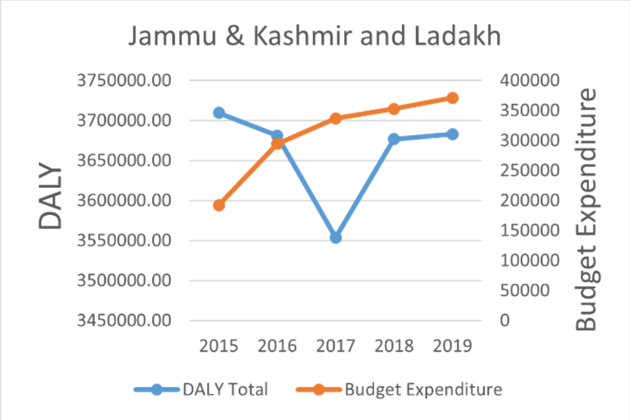
Jammu & Kashmir and Ladakh
DALY
3750000.00
3700000.00
3650000.00
3600000.00
3550000.00
3500000.00
3450000.00
400000
350000
300000
250000
200000
150000
100000
50000
0
Budget Expenditure
2015 2016 2017 2018 2019
DALY Total
Budget Expenditure

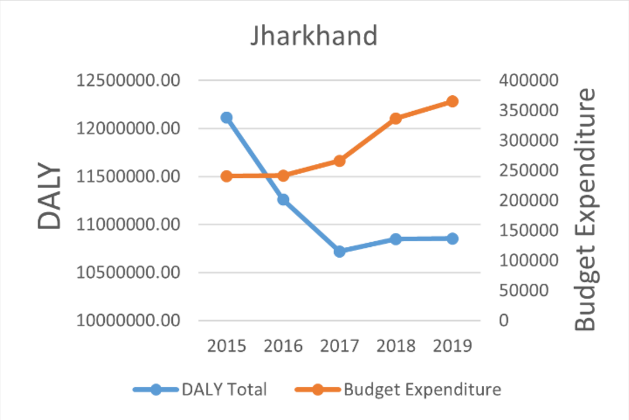
Jharkhand
DALY
12500000.00
12000000.00
11500000.00
11000000.00
10500000.00
10000000.00
400000
350000
300000
250000
200000
150000
100000
50000
0
Budget Expenditure
2015 2016 2017 2018 2019
DALY Total
Budget Expenditure

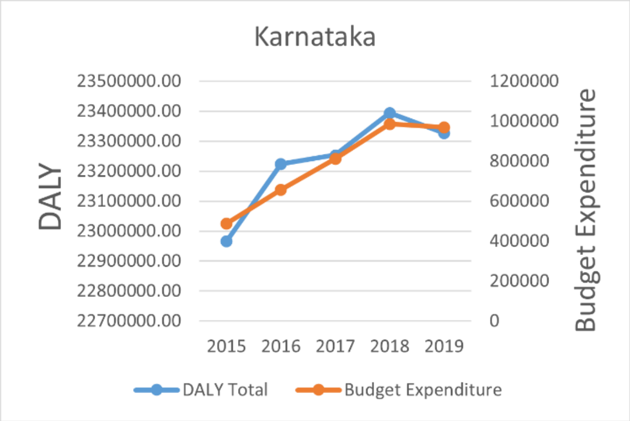
Karnataka
DALY
23500000.00
23400000.00
23300000.00
23200000.00
23100000.00
23000000.00
22900000.00
22800000.00
22700000.00
1200000
1000000
800000
600000
400000
200000
0
Budget Expenditure
2015 2016 2017 2018 2019
DALY Total
Budget Expenditure

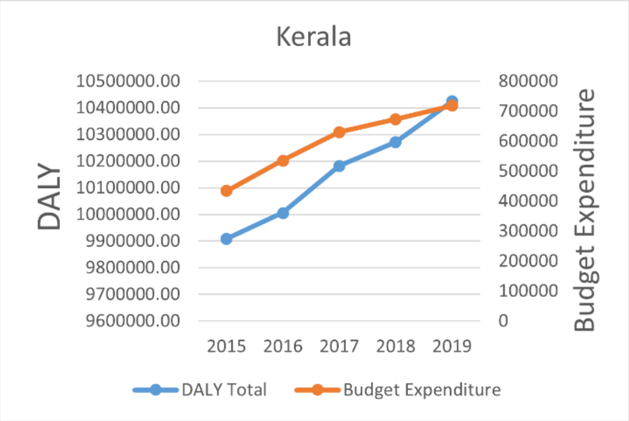
Kerala
DALY
10500000.00
10400000.00
10300000.00
10200000.00
10100000.00
10000000.00
9900000.00
9800000.00
9700000.00
9600000.00
800000
700000
600000
500000
400000
300000
200000
100000
0
Budget Expenditure
2015 2016 2017 2018 2019
DALY Total
Budget Expenditure

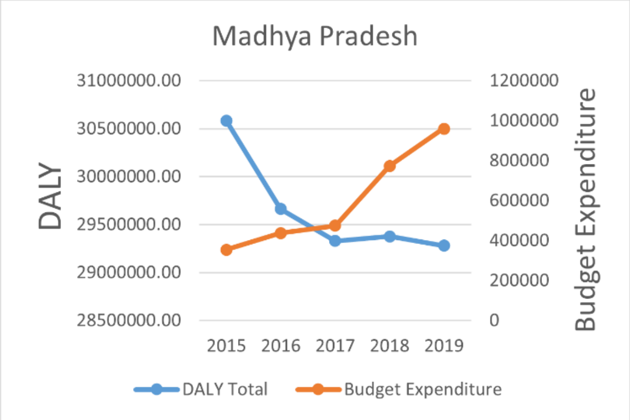
Madhya Pradesh
DALY
31000000.00
30500000.00
30000000.00
29500000.00
29000000.00
28500000.00
1200000
1000000
800000
600000
400000
200000
0
Budget Expenditure
2015 2016 2017 2018 2019
DALY Total
Budget Expenditure

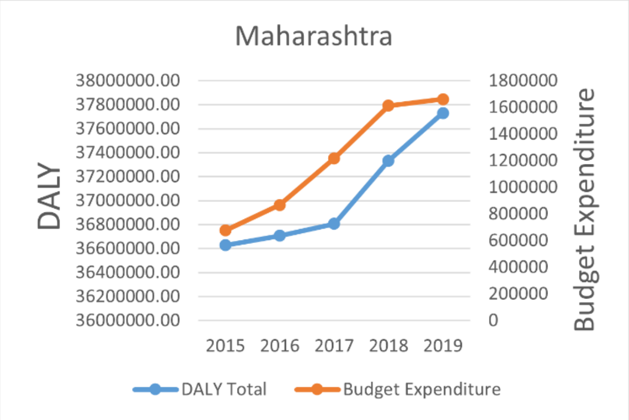
Maharashtra
DALY
38000000.00
37800000.00
37600000.00
37400000.00
37200000.00
37000000.00
36800000.00
36600000.00
36400000.00
36200000.00
36000000.00
1800000
1600000
1400000
1200000
1000000
800000
600000
400000
200000
0
Budget Expenditure
2015 2016 2017 2018 2019
DALY Total
Budget Expenditure

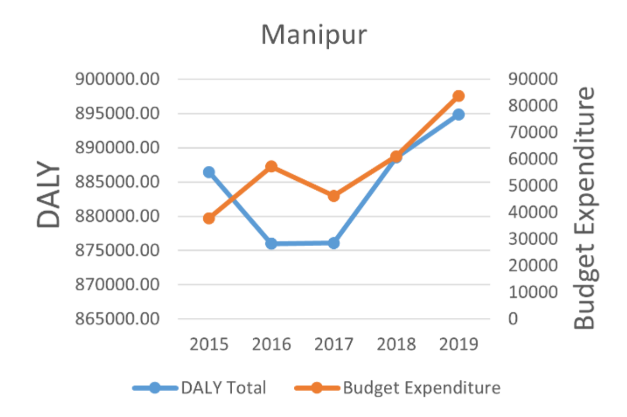
Manipur
900000.00
895000.00
890000.00
885000.00
880000.00
875000.00
870000.00
865000.00
DALY
90000
80000
70000
60000
50000
40000
30000
20000
10000
0
Budget Expenditure
2015 2016 2017 2018 2019
DALY Total
Budget Expenditure

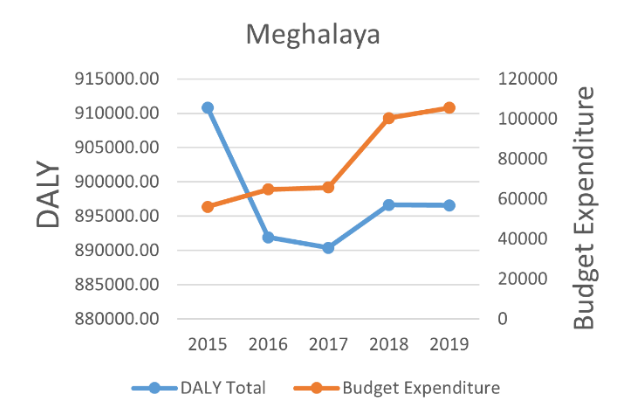
Meghalaya
915000.00
910000.00
905000.00
900000.00
895000.00
890000.00
885000.00
880000.00
DALY
120000
100000
80000
60000
40000
20000
0
Budget Expenditure
2015 2016 2017 2018 2019
DALY Total
Budget Expenditure

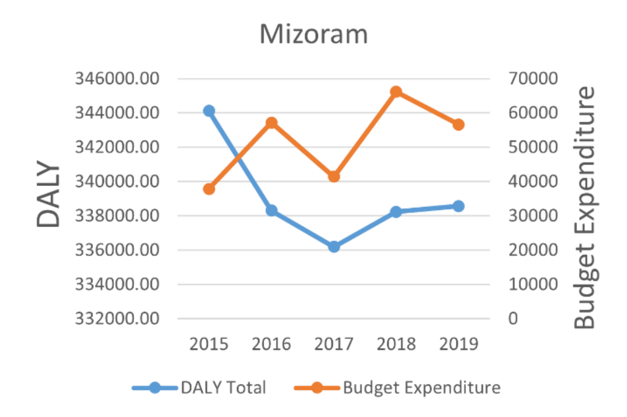
Mizoram
346000.00
344000.00
342000.00
340000.00
338000.00
336000.00
334000.00
332000.00
DALY
70000
60000
50000
40000
30000
20000
10000
0
Budget Expenditure
2015 2016 2017 2018 2019
DALY Total
Budget Expenditure

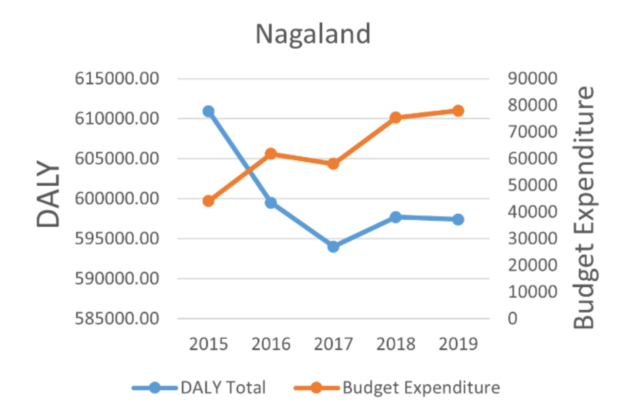
Nagaland
615000.00
610000.00
605000.00
600000.00
595000.00
590000.00
585000.00
DALY
90000
80000
70000
60000
50000
40000
30000
20000
10000
0
Budget Expenditure
2015 2016 2017 2018 2019
DALY Total
Budget Expenditure

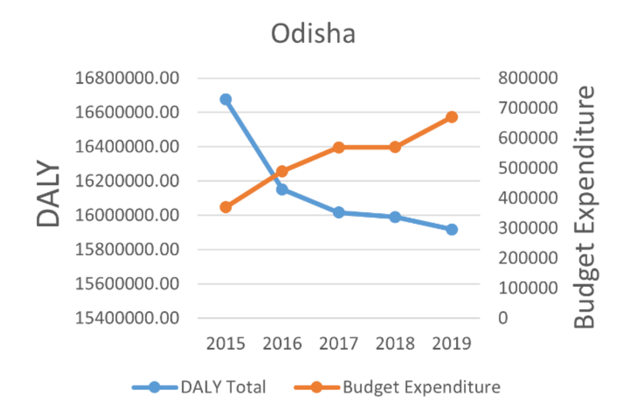
Odisha
16800000.00
16600000.00
16400000.00
16200000.00
16000000.00
15800000.00
15600000.00
15400000.00
DALY
800000
700000
600000
500000
400000
300000
200000
100000
0
Budget Expenditure
2015 2016 2017 2018 2019
DALY Total
Budget Expenditure

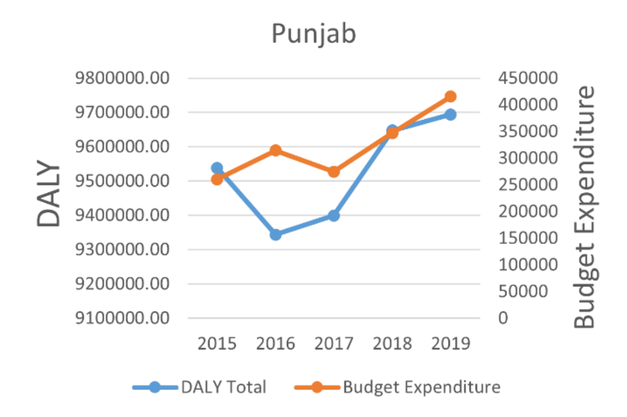
Punjab
9800000.00
9700000.00
9600000.00
9500000.00
9400000.00
9300000.00
9200000.00
9100000.00
DALY
450000
400000
350000
300000
250000
200000
150000
100000
50000
0
Budget Expenditure
2015 2016 2017 2018 2019
DALY Total
Budget Expenditure

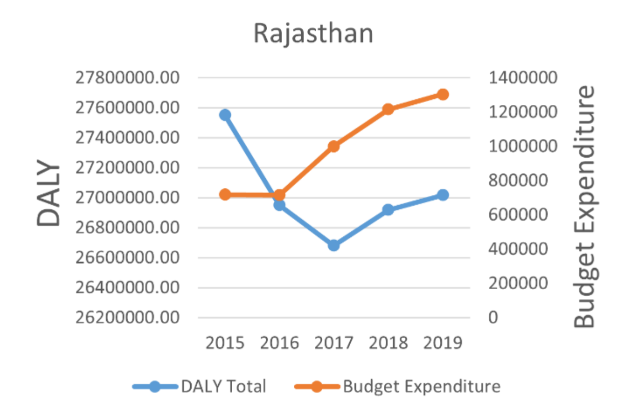
Rajasthan
27800000.00
27600000.00
27400000.00
27200000.00
27000000.00
26800000.00
26600000.00
26400000.00
26200000.00
DALY
1400000
1200000
1000000
800000
600000
400000
200000
0
Budget Expenditure
2015 2016 2017 2018 2019
DALY Total
Budget Expenditure

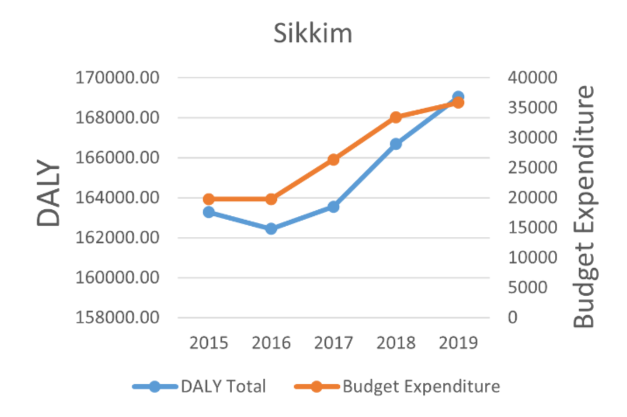
Sikkim
170000.00
168000.00
166000.00
164000.00
162000.00
160000.00
158000.00
DALY
40000
35000
30000
25000
20000
15000
10000
5000
0
Budget Expenditure
2015 2016 2017 2018 2019
DALY Total
Budget Expenditure

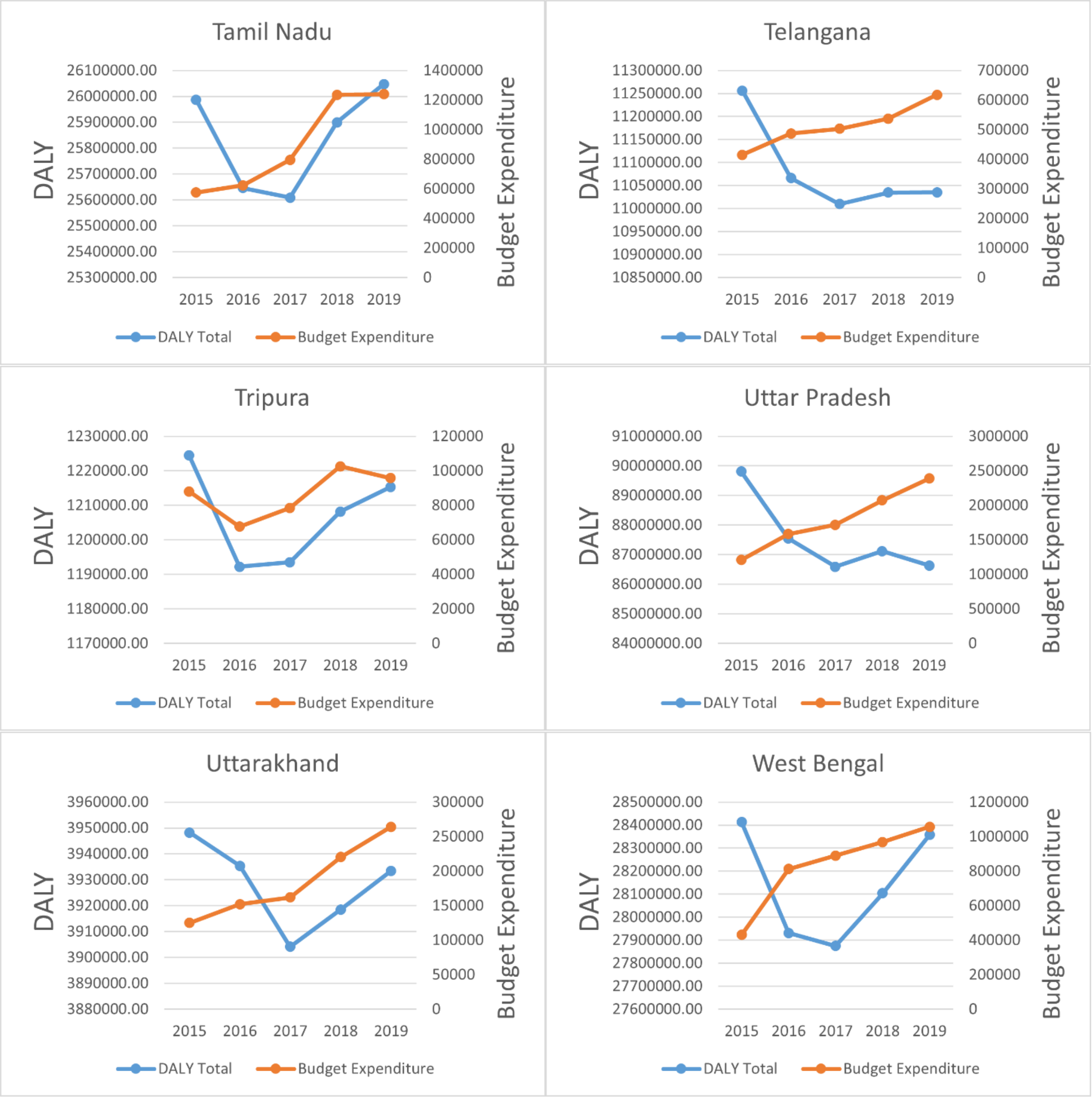

[Line graph with the primary Y-axis represents total DALY from 2015 to 2019 for individual states in India. Secondary Y-axis represents yearly budget during the same period for individual states]

Appendix III. Change in disease burden due to Non-Communicable Diseases and budget allocation in Indian states

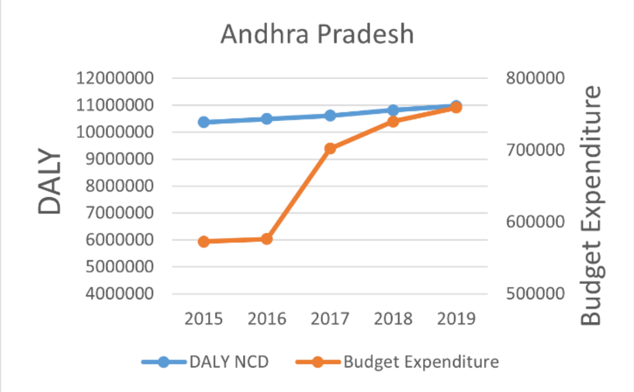

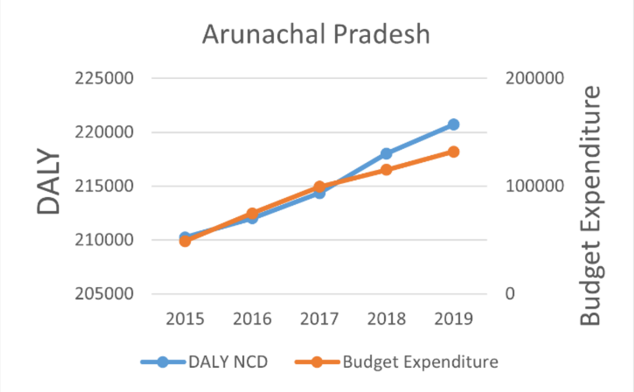

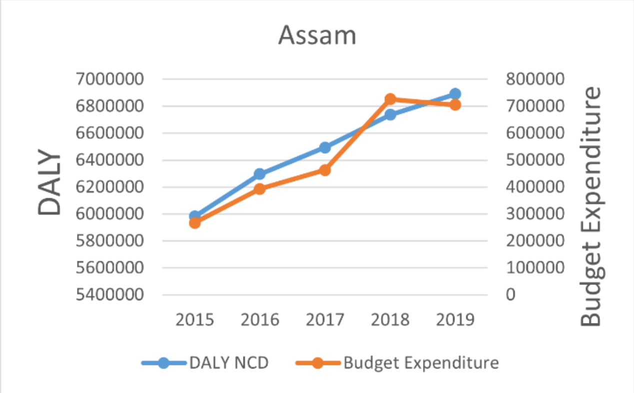

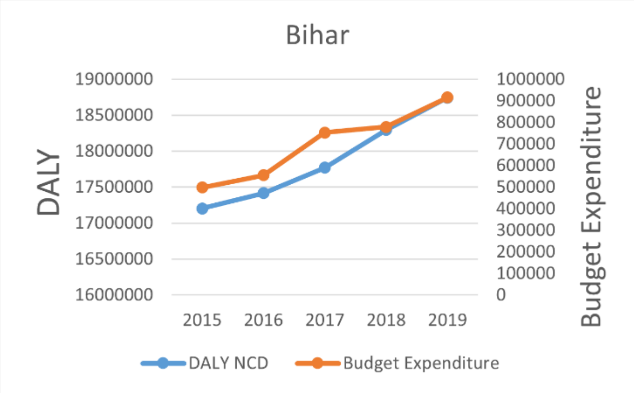

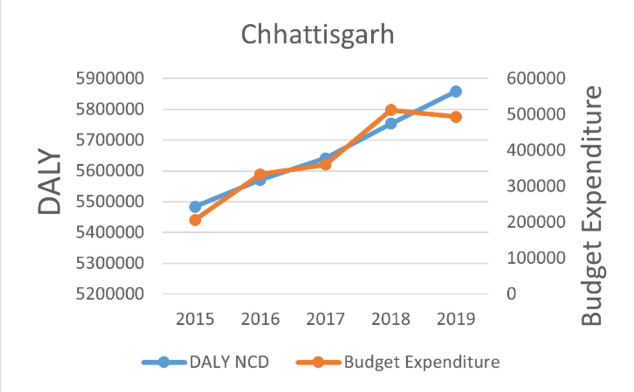

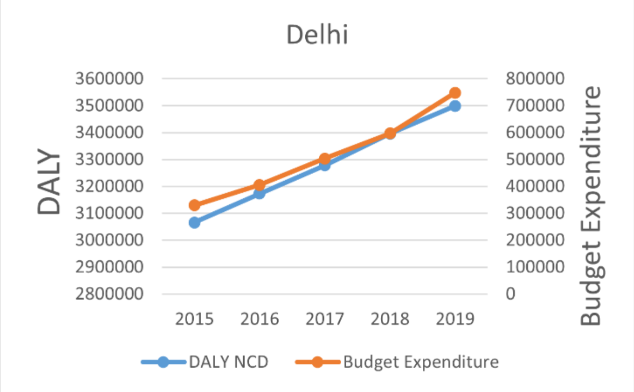

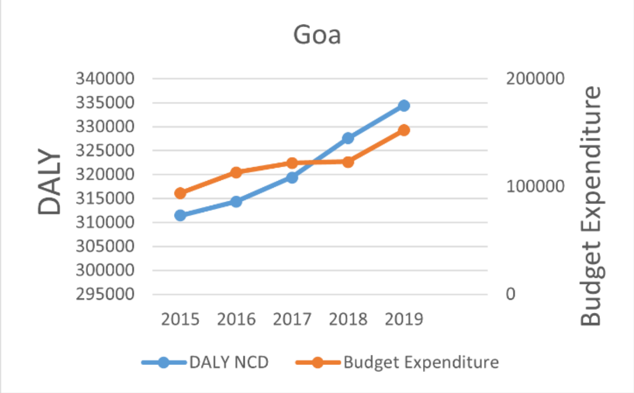

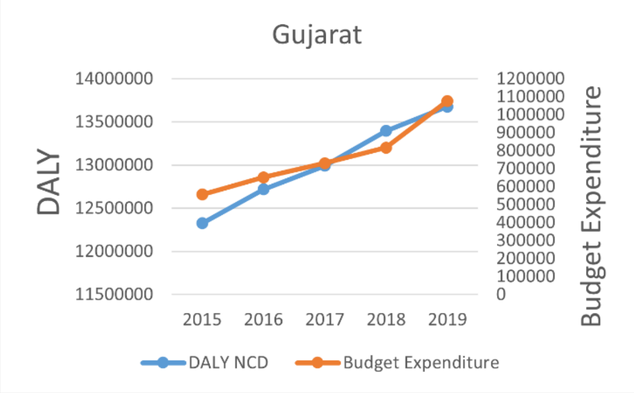

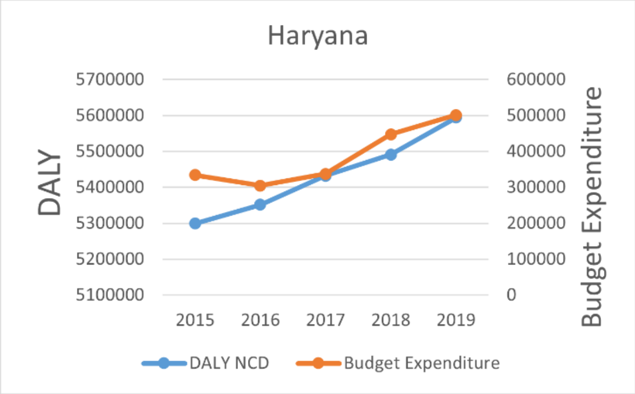
Haryana
DALY
5700000
5600000
5500000
5400000
5300000
5200000
5100000
Budget Expenditure
600000
500000
400000
300000
200000
100000
0
2015 2016 2017 2018 2019
DALY NCD
Budget Expenditure

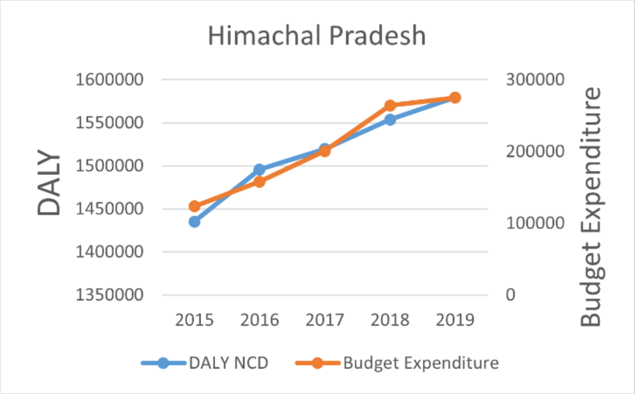
Himachal Pradesh
DALY
1600000
1550000
1500000
1450000
1400000
1350000
Budget Expenditure
300000
200000
100000
0
2015 2016 2017 2018 2019
DALY NCD
Budget Expenditure

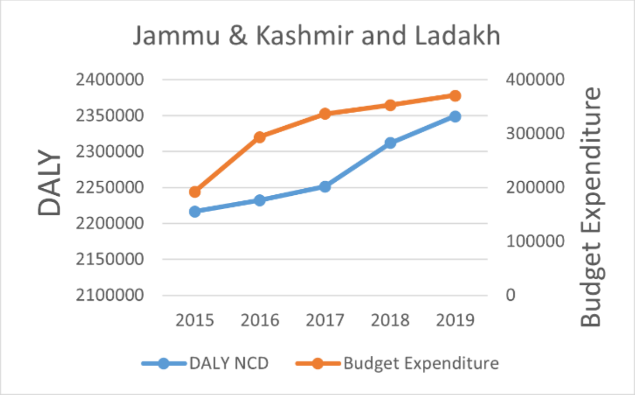
Jammu & Kashmir and Ladakh
DALY
2400000
2350000
2300000
2250000
2200000
2150000
2100000
Budget Expenditure
400000
300000
200000
100000
0
2015 2016 2017 2018 2019
DALY NCD
Budget Expenditure

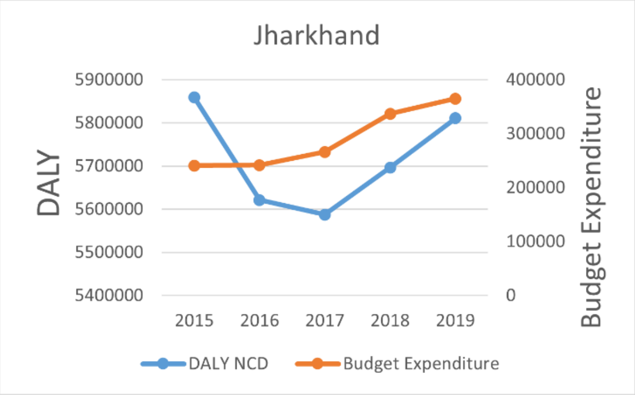
Jharkhand
DALY
5900000
5800000
5700000
5600000
5500000
5400000
Budget Expenditure
400000
300000
200000
100000
0
2015 2016 2017 2018 2019
DALY NCD
Budget Expenditure

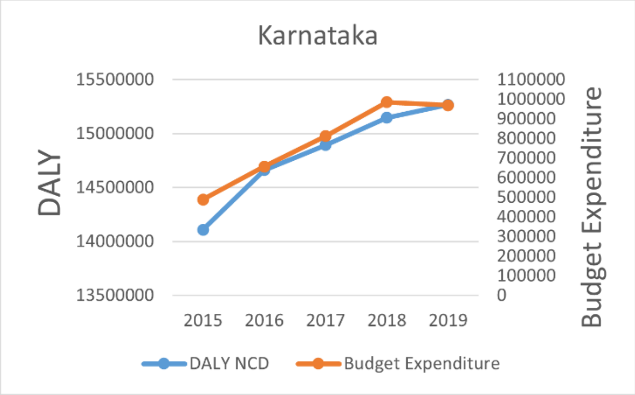
Karnataka
DALY
15500000
15000000
14500000
14000000
13500000
Budget Expenditure
1100000
1000000
900000
800000
700000
600000
500000
400000
300000
200000
100000
0
2015 2016 2017 2018 2019
DALY NCD
Budget Expenditure

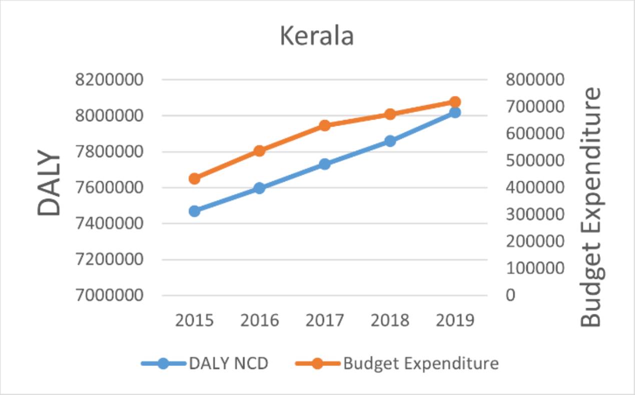
Kerala
DALY
8200000
8000000
7800000
7600000
7400000
7200000
7000000
Budget Expenditure
800000
700000
600000
500000
400000
300000
200000
100000
0
2015 2016 2017 2018 2019
DALY NCD
Budget Expenditure

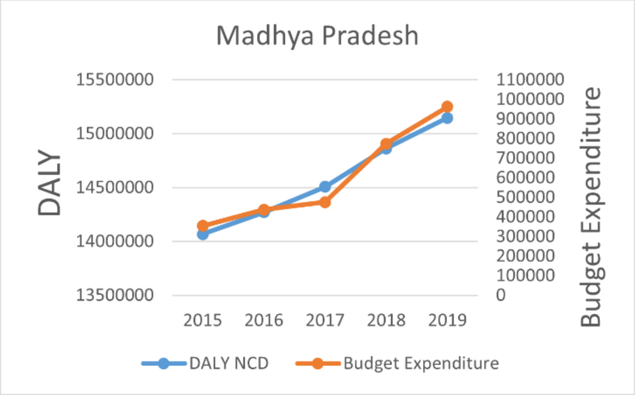
Madhya Pradesh
DALY
15500000
15000000
14500000
14000000
13500000
Budget Expenditure
1100000
1000000
900000
800000
700000
600000
500000
400000
300000
200000
100000
0
2015 2016 2017 2018 2019
DALY NCD
Budget Expenditure

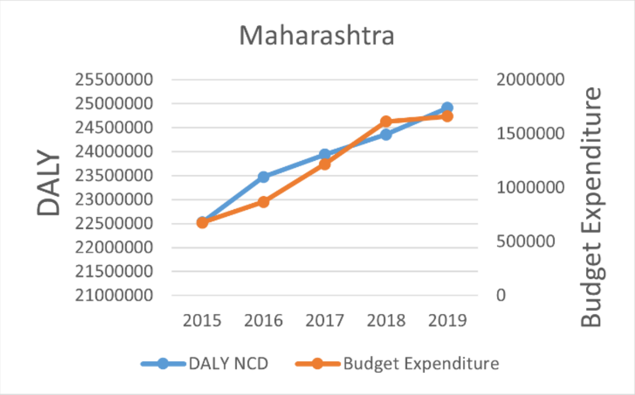
Maharashtra
DALY
25500000
25000000
24500000
24000000
23500000
23000000
22500000
22000000
21500000
21000000
Budget Expenditure
2000000
1500000
1000000
500000
0
2015 2016 2017 2018 2019
DALY NCD
Budget Expenditure

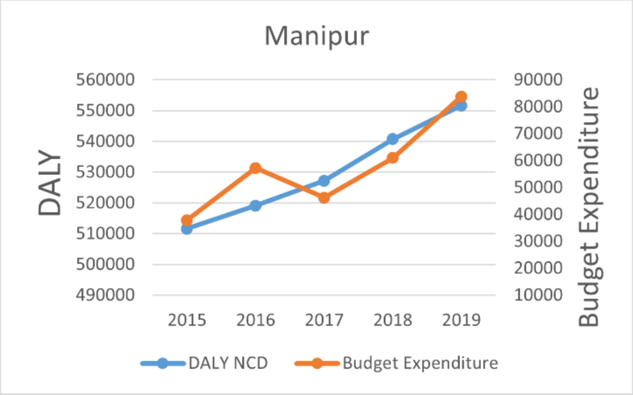
Manipur
DALY
560000
550000
540000
530000
520000
510000
500000
490000
90000
80000
70000
60000
50000
40000
30000
20000
10000
Budget Expenditure
2015
2016
2017
2018
2019
DALY NCD
Budget Expenditure

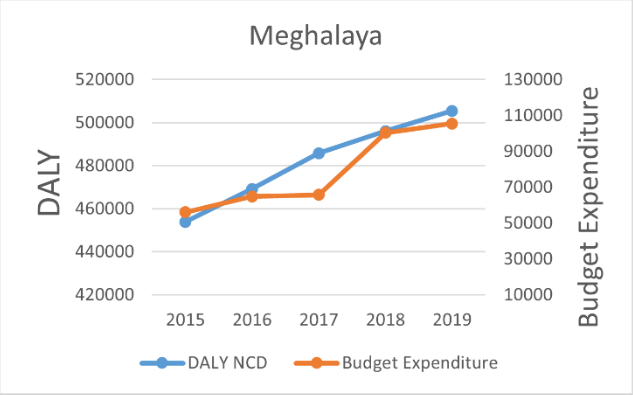
Meghalaya
DALY
520000
500000
480000
460000
440000
420000
130000
110000
90000
70000
50000
30000
10000
Budget Expenditure
2015
2016
2017
2018
2019
DALY NCD
Budget Expenditure

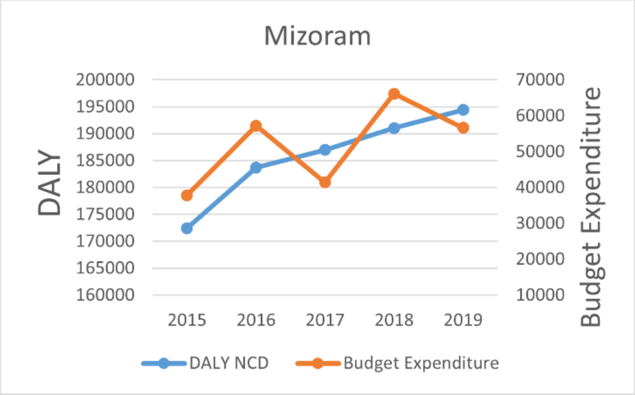
Mizoram
DALY
200000
195000
190000
185000
180000
175000
170000
165000
160000
70000
60000
50000
40000
30000
20000
10000
Budget Expenditure
2015
2016
2017
2018
2019
DALY NCD
Budget Expenditure

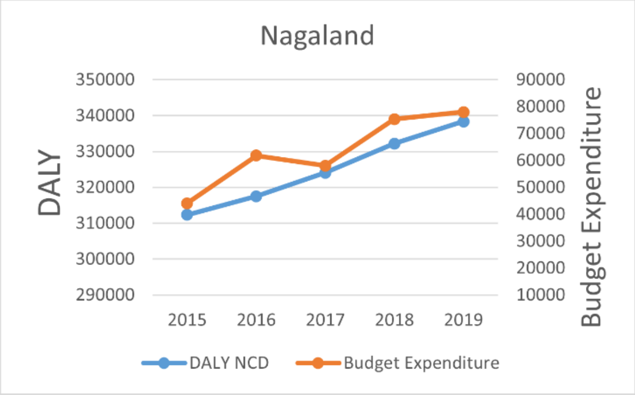
Nagaland
DALY
350000
340000
330000
320000
310000
300000
290000
90000
80000
70000
60000
50000
40000
30000
20000
10000
Budget Expenditure
2015
2016
2017
2018
2019
DALY NCD
Budget Expenditure

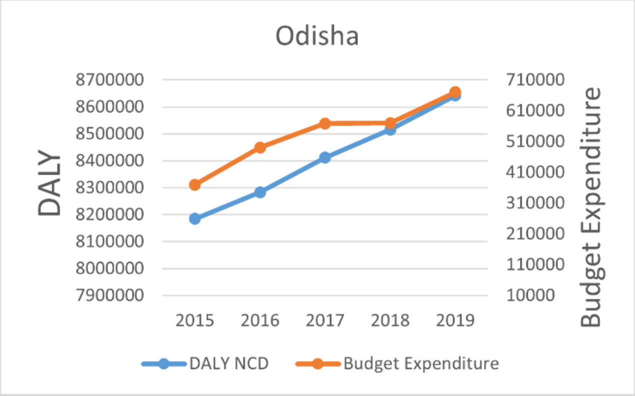
Odisha
DALY
8700000
8600000
8500000
8400000
8300000
8200000
8100000
8000000
7900000
710000
610000
510000
410000
310000
210000
110000
10000
Budget Expenditure
2015
2016
2017
2018
2019
DALY NCD
Budget Expenditure

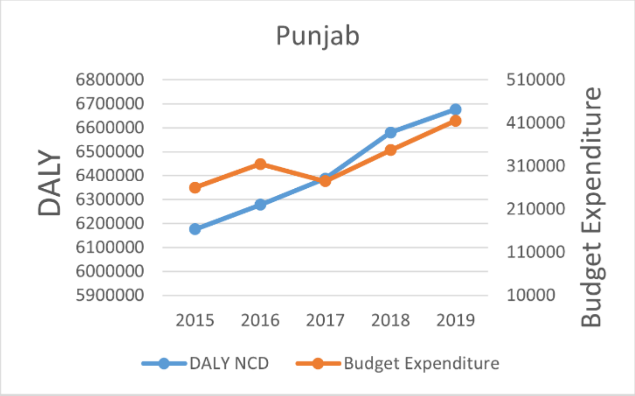
Punjab
DALY
6800000
6700000
6600000
6500000
6400000
6300000
6200000
6100000
6000000
5900000
510000
410000
310000
210000
110000
10000
Budget Expenditure
2015
2016
2017
2018
2019
DALY NCD
Budget Expenditure

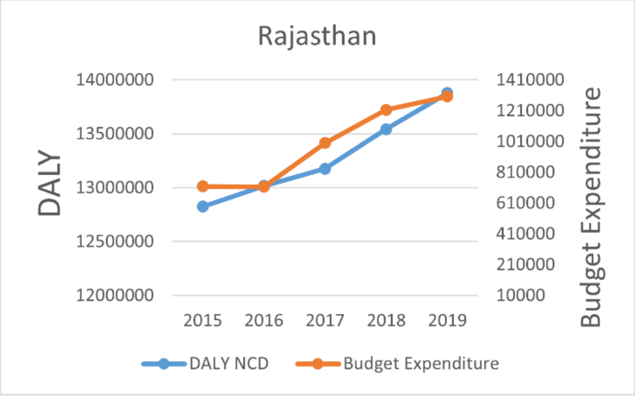
Rajasthan
DALY
14000000
13500000
13000000
12500000
12000000
1410000
1210000
1010000
810000
610000
410000
210000
10000
Budget Expenditure
2015
2016
2017
2018
2019
DALY NCD
Budget Expenditure

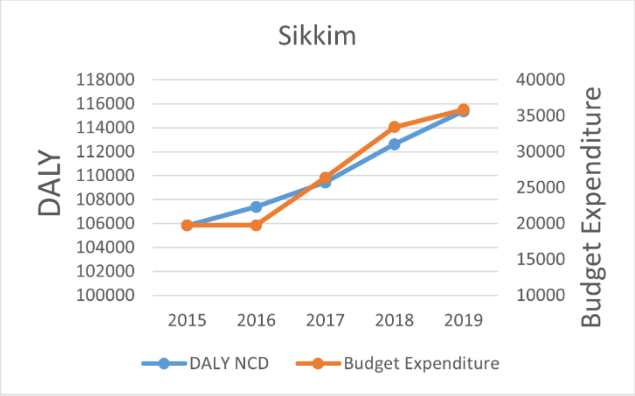
Sikkim
DALY
118000
116000
114000
112000
110000
108000
106000
104000
102000
100000
40000
35000
30000
25000
20000
15000
10000
Budget Expenditure
2015
2016
2017
2018
2019
DALY NCD
Budget Expenditure

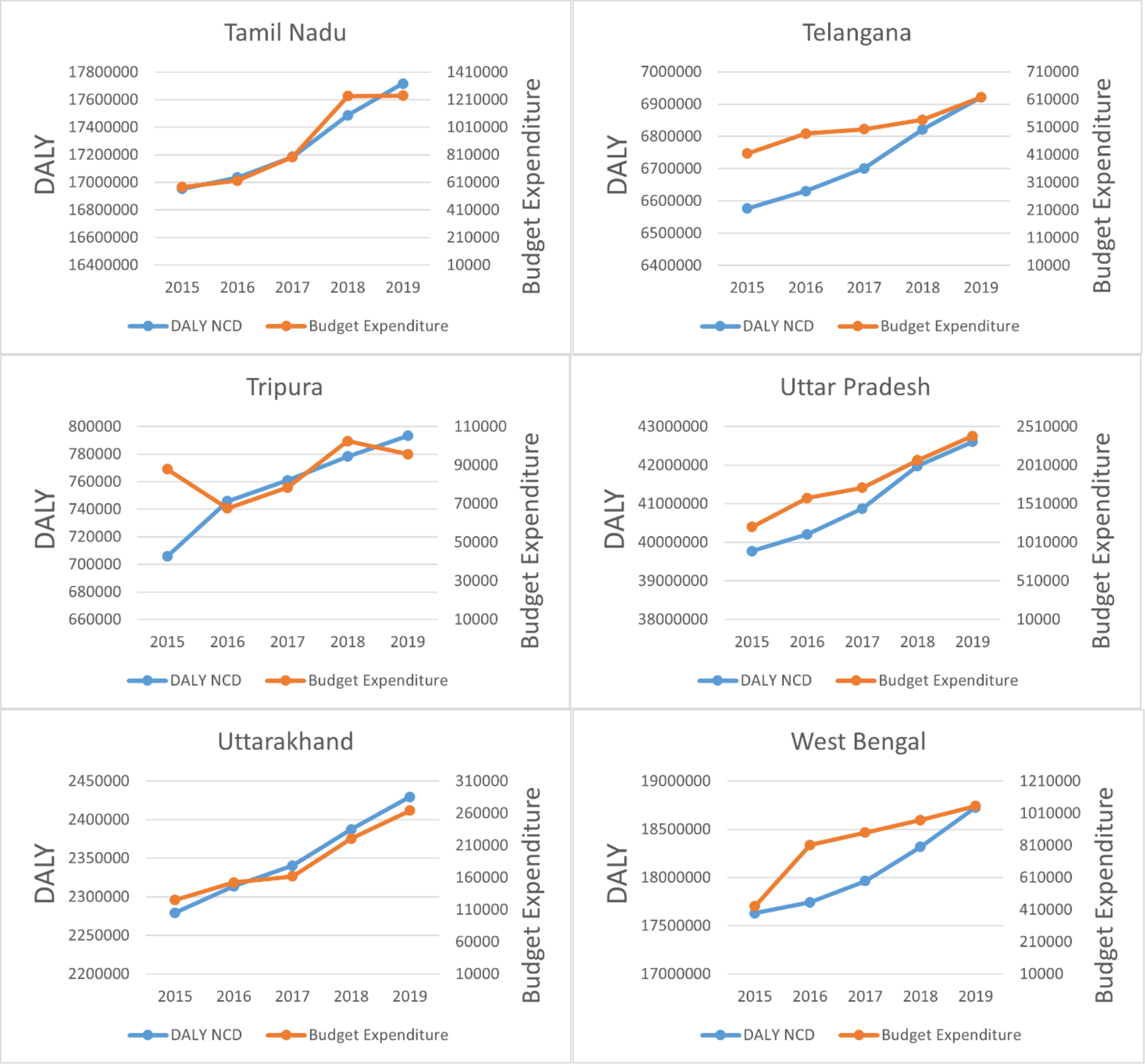

[Line graph with the primary Y-axis represents total DALY due to NCDs from 2015 to 2019 for individual states in India. Secondary Y-axis represents yearly budget during the same period for individual states in India]

Appendix IV. Change in disease burden due to Communicable Diseases and budget allocation in Indian states

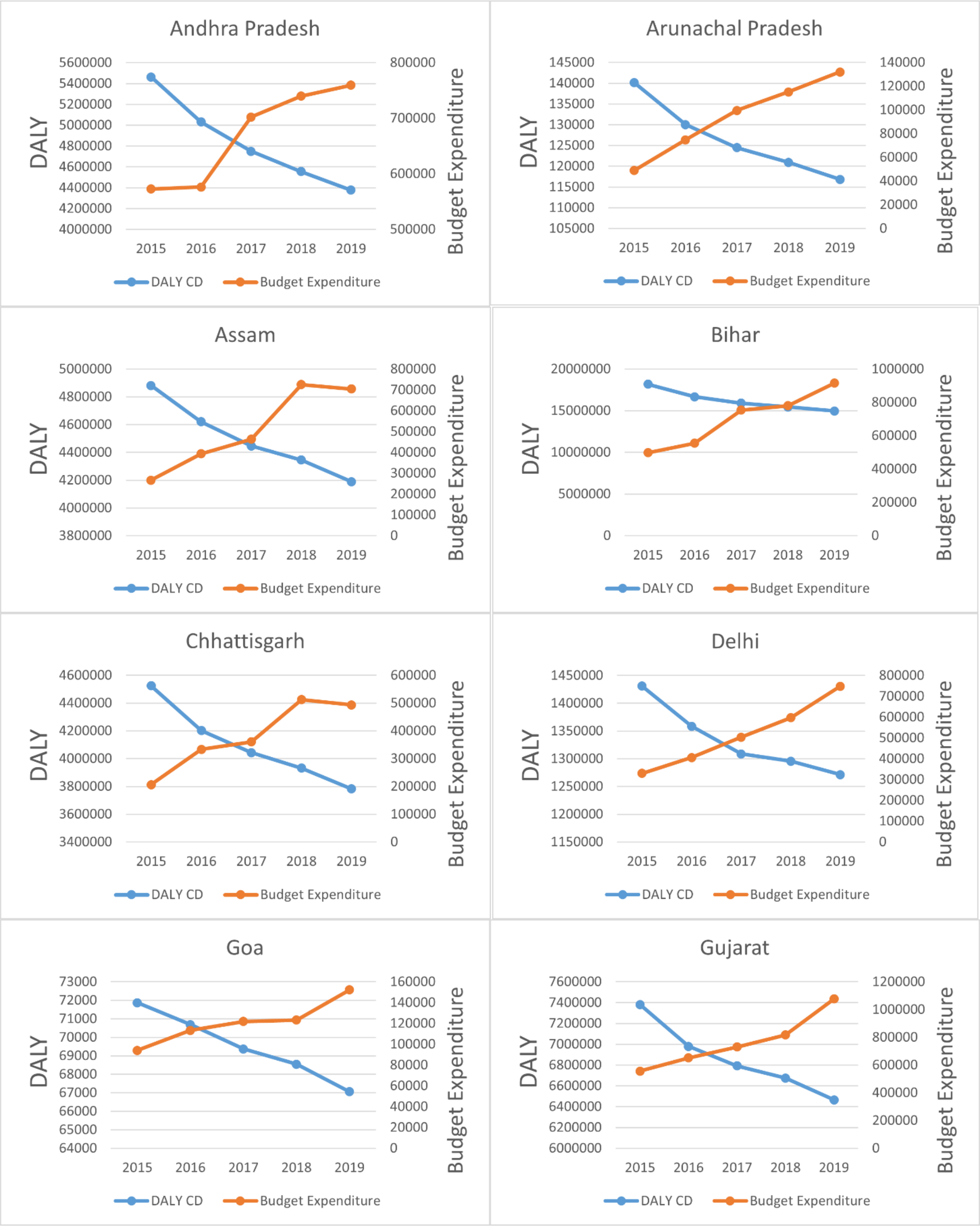

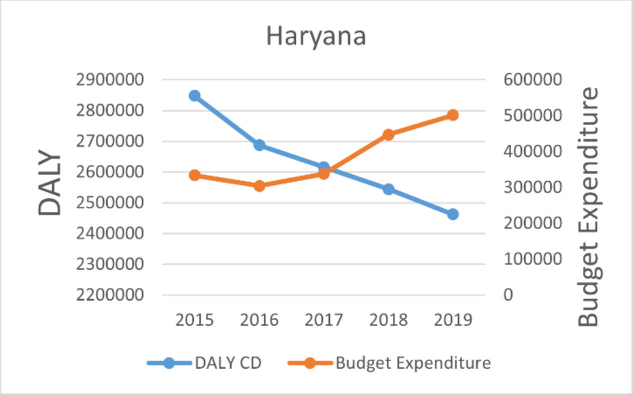
Haryana
DALY
2900000
2800000
2700000
2600000
2500000
2400000
2300000
2200000
600000
500000
400000
300000
200000
100000
0
Budget Expenditure
2015
2016
2017
2018
2019
DALY CD
Budget Expenditure

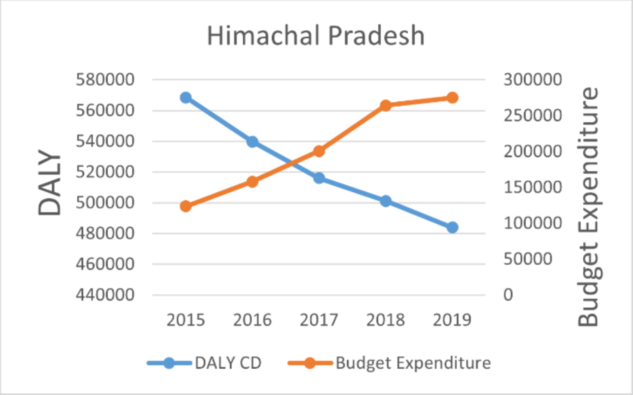
Himachal Pradesh
DALY
580000
560000
540000
520000
500000
480000
460000
440000
300000
250000
200000
150000
100000
50000
0
Budget Expenditure
2015
2016
2017
2018
2019
DALY CD
Budget Expenditure

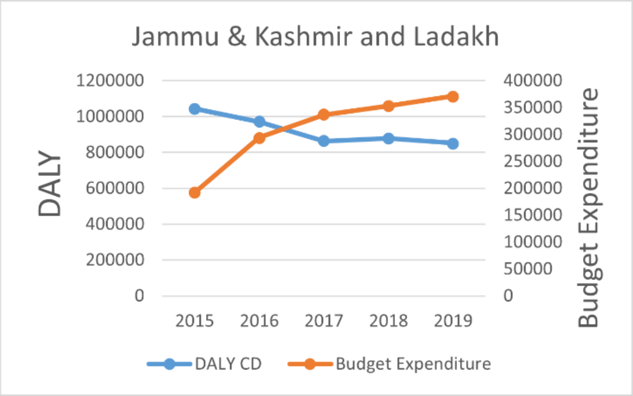
Jammu & Kashmir and Ladakh
DALY
1200000
1000000
800000
600000
400000
200000
0
400000
350000
300000
250000
200000
150000
100000
50000
0
Budget Expenditure
2015
2016
2017
2018
2019
DALY CD
Budget Expenditure

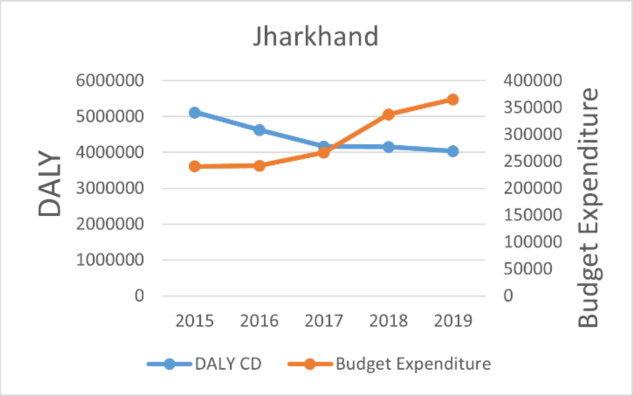
Jharkhand
DALY
6000000
5000000
4000000
3000000
2000000
1000000
0
400000
350000
300000
250000
200000
150000
100000
50000
0
Budget Expenditure
2015
2016
2017
2018
2019
DALY CD
Budget Expenditure

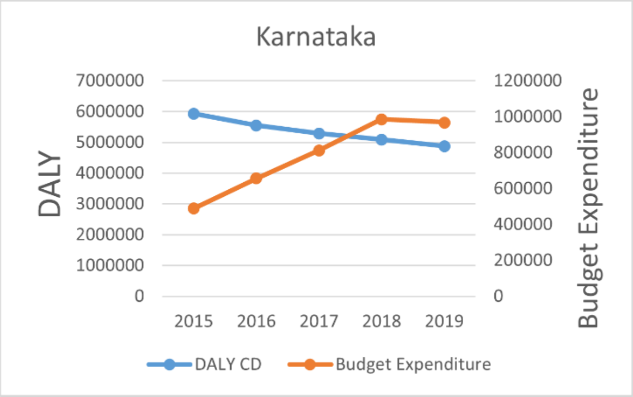
Karnataka
DALY
7000000
6000000
5000000
4000000
3000000
2000000
1000000
0
1200000
1000000
800000
600000
400000
200000
0
Budget Expenditure
2015
2016
2017
2018
2019
DALY CD
Budget Expenditure

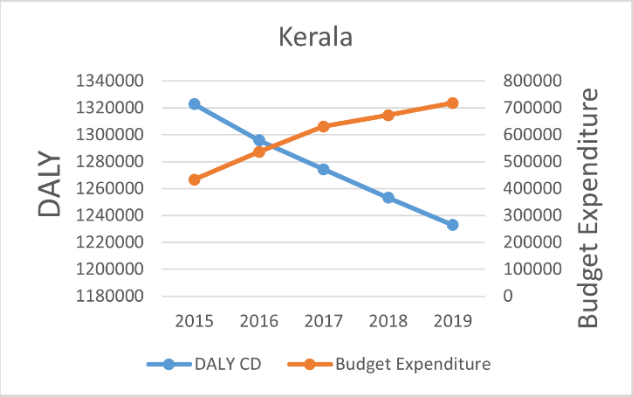
Kerala
DALY
1340000
1320000
1300000
1280000
1260000
1240000
1220000
1200000
1180000
800000
700000
600000
500000
400000
300000
200000
100000
0
Budget Expenditure
2015
2016
2017
2018
2019
DALY CD
Budget Expenditure

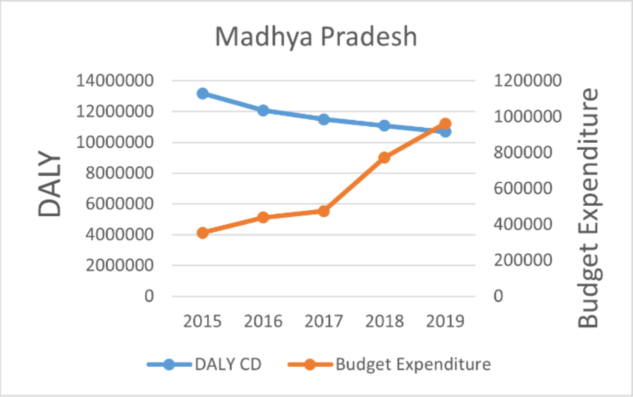
Madhya Pradesh
DALY
14000000
12000000
10000000
8000000
6000000
4000000
2000000
0
1200000
1000000
800000
600000
400000
200000
0
Budget Expenditure
2015
2016
2017
2018
2019
DALY CD
Budget Expenditure

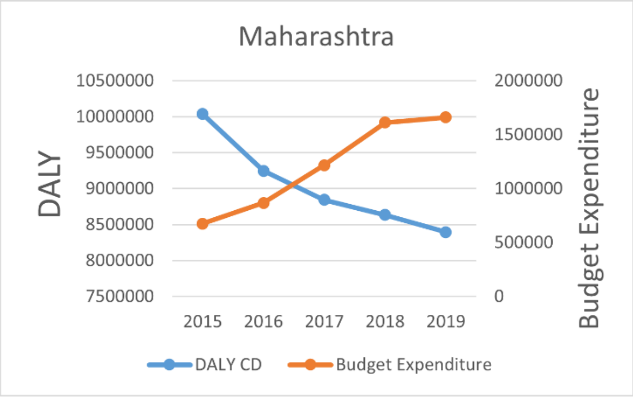
Maharashtra
DALY
10500000
10000000
9500000
9000000
8500000
8000000
7500000
2000000
1500000
1000000
500000
0
Budget Expenditure
2015
2016
2017
2018
2019
DALY CD
Budget Expenditure

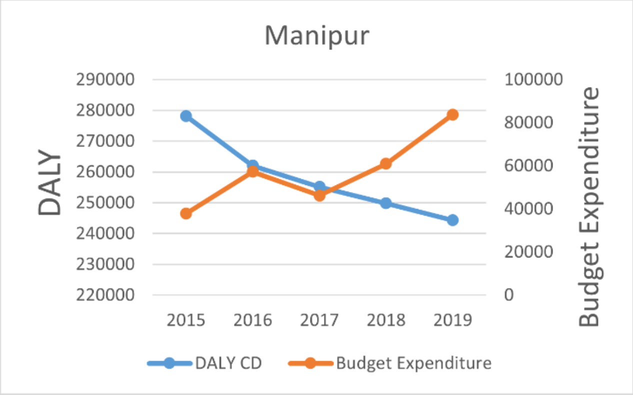
Manipur
DALY
290000
280000
270000
260000
250000
240000
230000
220000
100000
80000
60000
40000
20000
0
Budget Expenditure
2015 2016 2017 2018 2019
DALY CD
Budget Expenditure

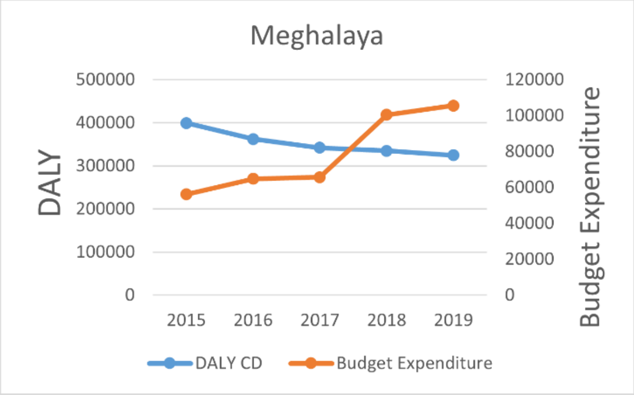
Meghalaya
DALY
500000
400000
300000
200000
100000
0
120000
100000
80000
60000
40000
20000
0
Budget Expenditure
2015 2016 2017 2018 2019
DALY CD
Budget Expenditure

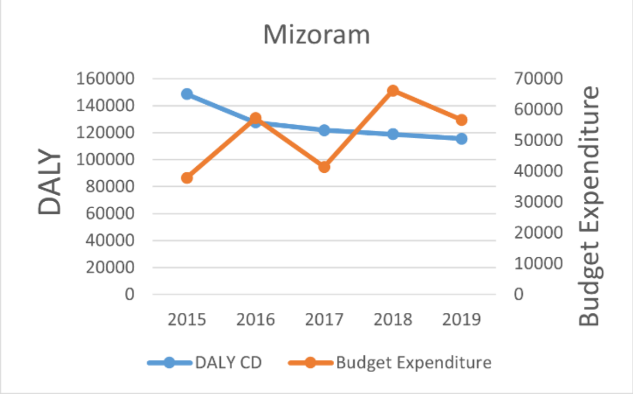
Mizoram
DALY
160000
140000
120000
100000
80000
60000
40000
20000
0
70000
60000
50000
40000
30000
20000
10000
0
Budget Expenditure
2015 2016 2017 2018 2019
DALY CD
Budget Expenditure

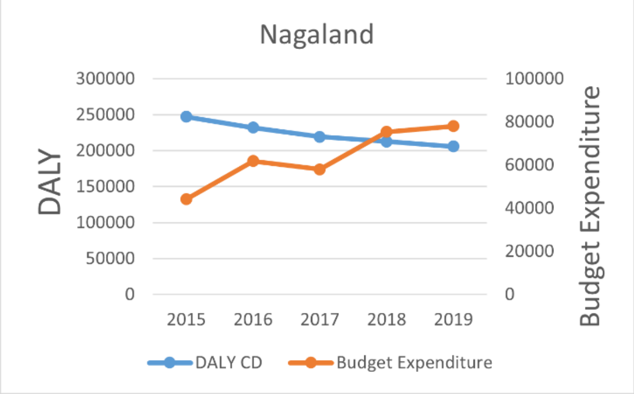
Nagaland
DALY
300000
250000
200000
150000
100000
50000
0
100000
80000
60000
40000
20000
0
Budget Expenditure
2015 2016 2017 2018 2019
DALY CD
Budget Expenditure

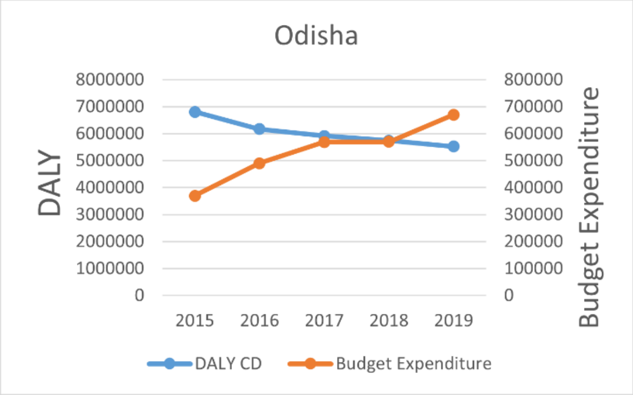
Odisha
DALY
8000000
7000000
6000000
5000000
4000000
3000000
2000000
1000000
0
800000
700000
600000
500000
400000
300000
200000
100000
0
Budget Expenditure
2015 2016 2017 2018 2019
DALY CD
Budget Expenditure

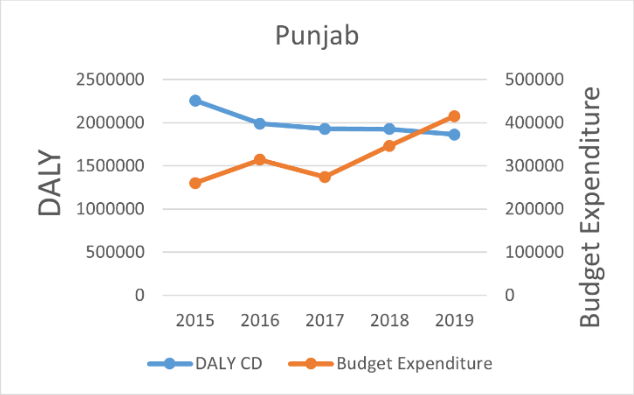
Punjab
DALY
2500000
2000000
1500000
1000000
500000
0
500000
400000
300000
200000
100000
0
Budget Expenditure
2015 2016 2017 2018 2019
DALY CD
Budget Expenditure

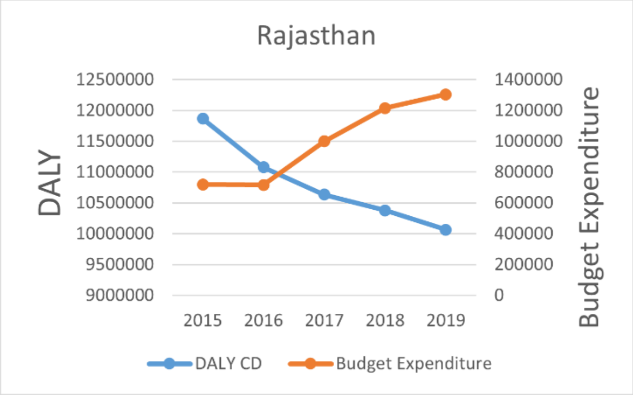
Rajasthan
DALY
12500000
12000000
11500000
11000000
10500000
10000000
9500000
9000000
1400000
1200000
1000000
800000
600000
400000
200000
0
Budget Expenditure
2015 2016 2017 2018 2019
DALY CD
Budget Expenditure

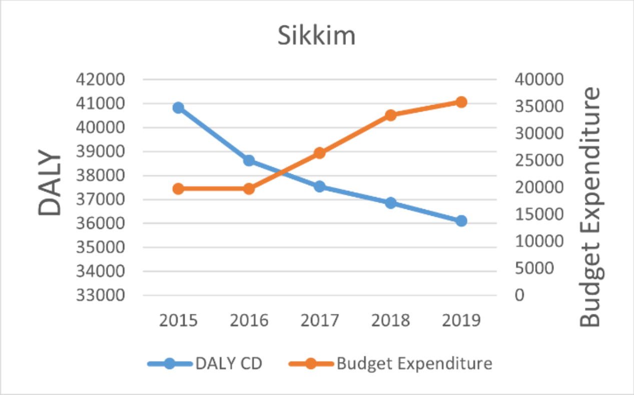
Sikkim
DALY
42000
41000
40000
39000
38000
37000
36000
35000
34000
33000
40000
35000
30000
25000
20000
15000
10000
5000
0
Budget Expenditure
2015 2016 2017 2018 2019
DALY CD
Budget Expenditure

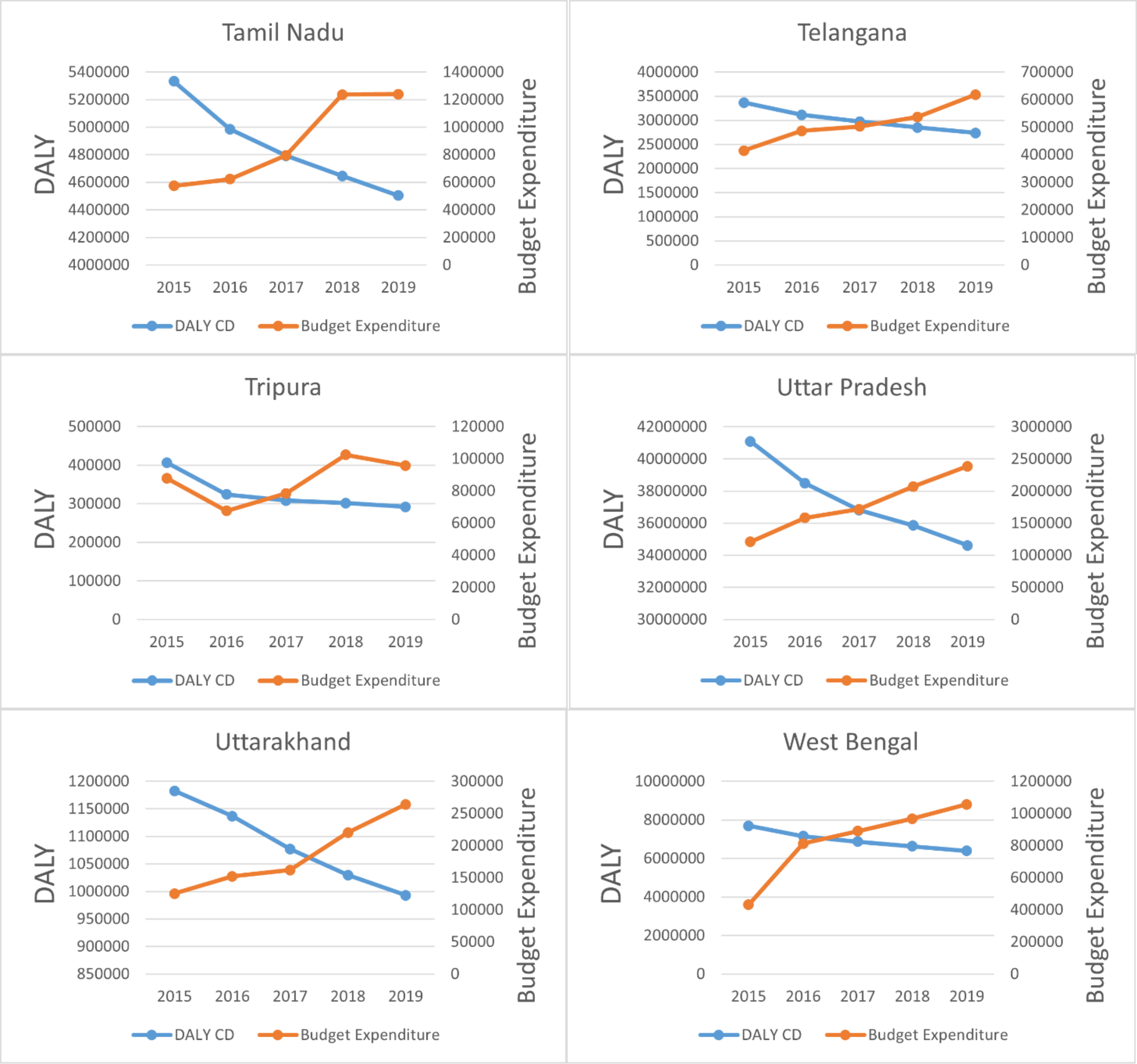

[Line graph with the primary Y-axis represents total DALY due to CDs from 2015 to 2019 for individual states in India. Secondary Y-axis represents yearly budget during the same period for individual states in India]

Appendix V. Change in disease burden due to Injuries and budget allocation in Indian states

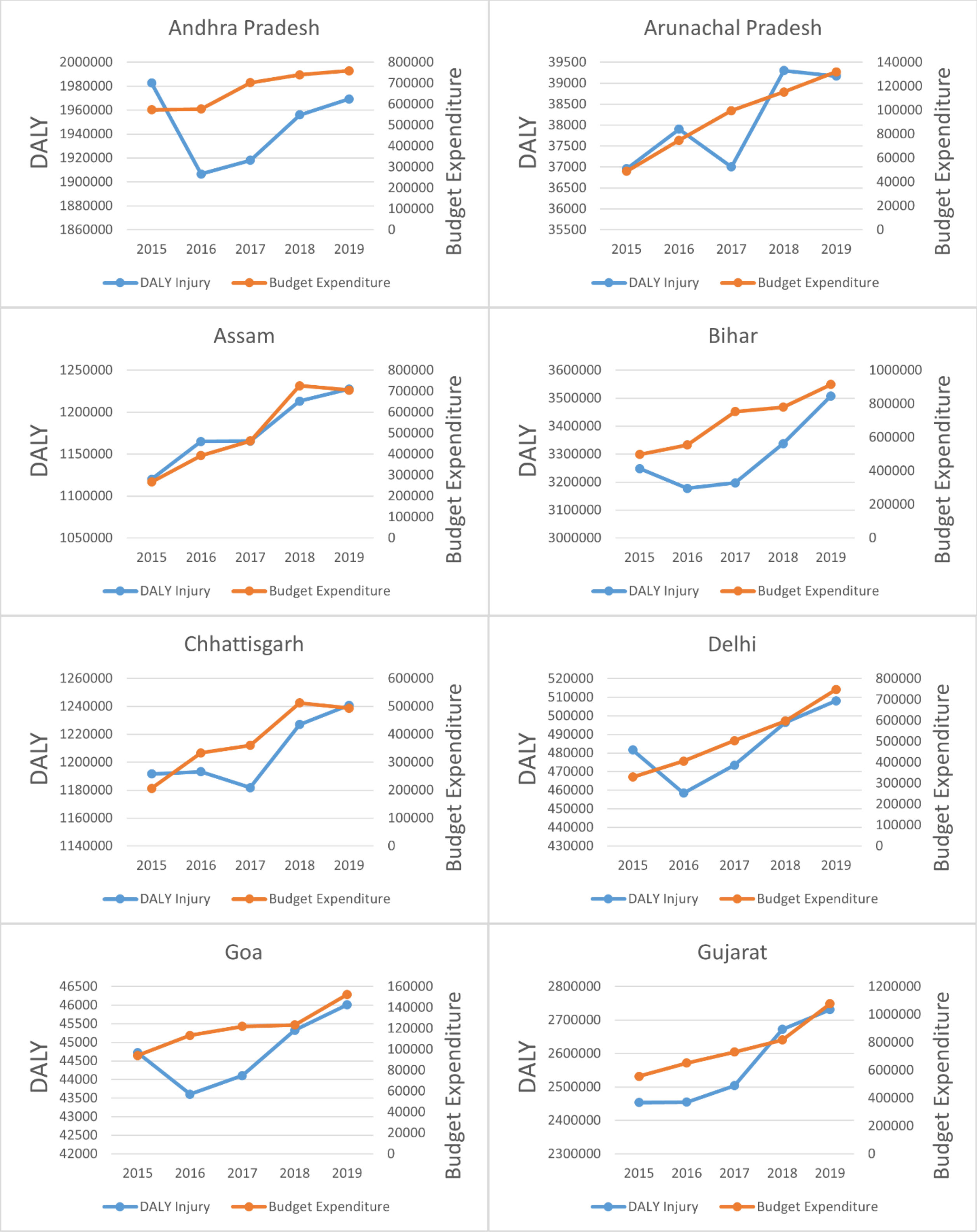

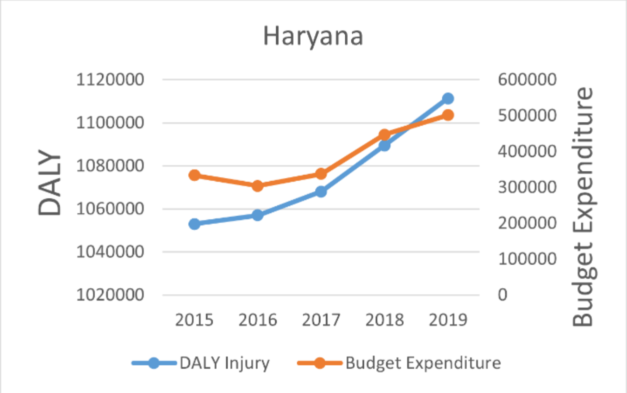
Haryana
DALY
1120000
1100000
1080000
1060000
1040000
1020000
600000
500000
400000
300000
200000
100000
0
Budget Expenditure
2015
2016
2017
2018
2019
DALY Injury
Budget Expenditure

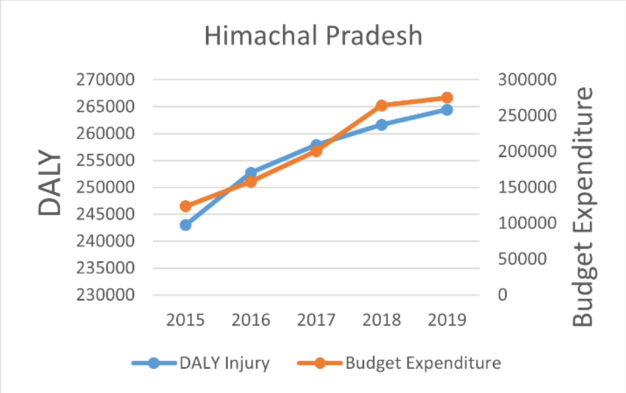
Himachal Pradesh
DALY
270000
265000
260000
255000
250000
245000
240000
235000
230000
300000
250000
200000
150000
100000
50000
0
Budget Expenditure
2015
2016
2017
2018
2019
DALY Injury
Budget Expenditure

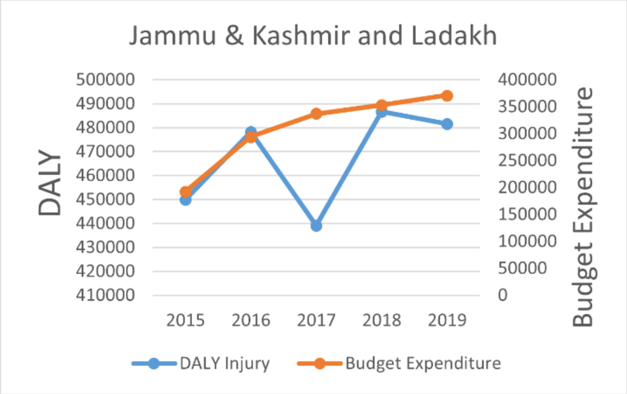
Jammu & Kashmir and Ladakh
DALY
500000
490000
480000
470000
460000
450000
440000
430000
420000
410000
400000
350000
300000
250000
200000
150000
100000
50000
0
Budget Expenditure
2015
2016
2017
2018
2019
DALY Injury
Budget Expenditure

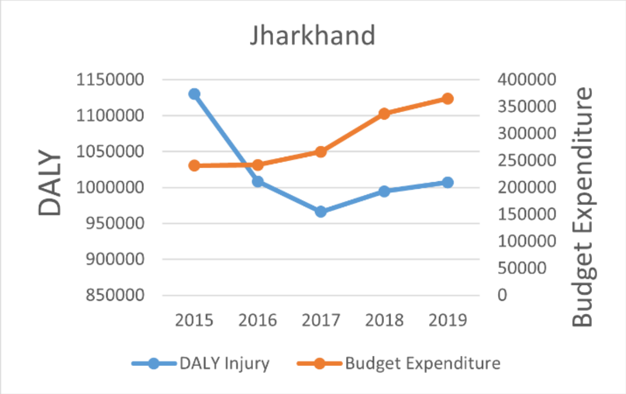
Jharkhand
DALY
1150000
1100000
1050000
1000000
950000
900000
850000
400000
350000
300000
250000
200000
150000
100000
50000
0
Budget Expenditure
2015
2016
2017
2018
2019
DALY Injury
Budget Expenditure

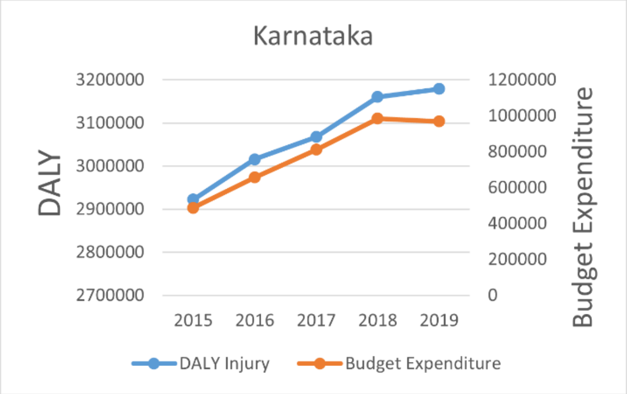
Karnataka
DALY
3200000
3100000
3000000
2900000
2800000
2700000
1200000
1000000
800000
600000
400000
200000
0
Budget Expenditure
2015
2016
2017
2018
2019
DALY Injury
Budget Expenditure

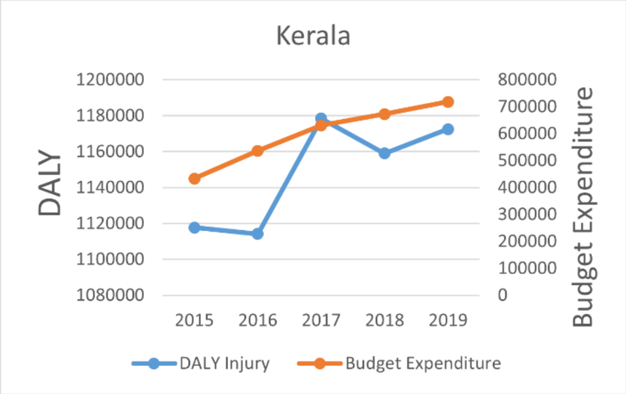
Kerala
DALY
1200000
1180000
1160000
1140000
1120000
1100000
1080000
800000
700000
600000
500000
400000
300000
200000
100000
0
Budget Expenditure
2015
2016
2017
2018
2019
DALY Injury
Budget Expenditure

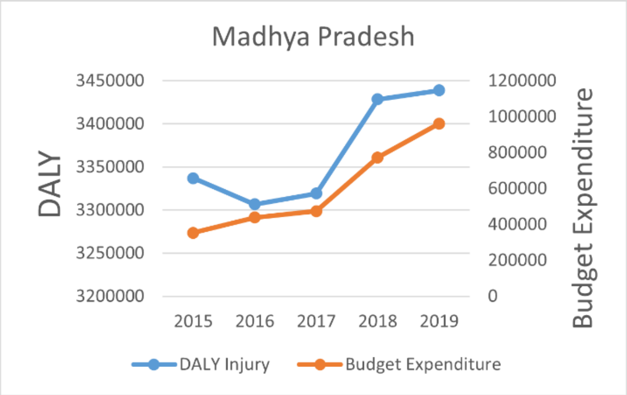
Madhya Pradesh
DALY
3450000
3400000
3350000
3300000
3250000
3200000
1200000
1000000
800000
600000
400000
200000
0
Budget Expenditure
2015
2016
2017
2018
2019
DALY Injury
Budget Expenditure

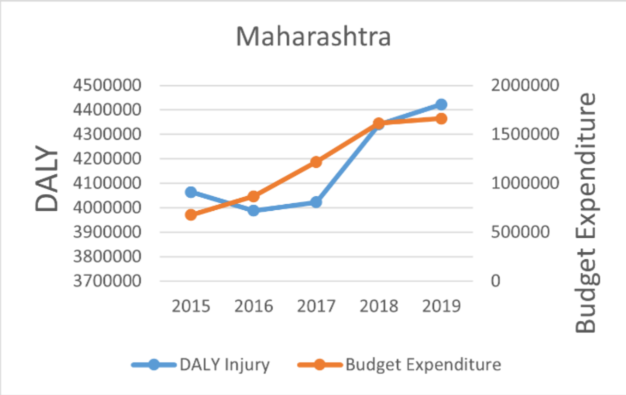
Maharashtra
DALY
4500000
4400000
4300000
4200000
4100000
4000000
3900000
3800000
3700000
2000000
1500000
1000000
500000
0
Budget Expenditure
2015
2016
2017
2018
2019
DALY Injury
Budget Expenditure

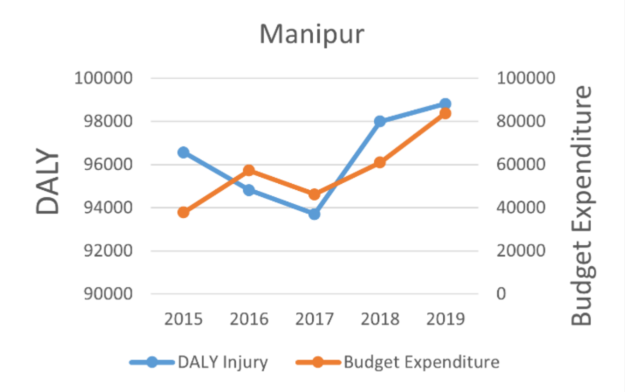
Manipur
DALY
100000
98000
96000
94000
92000
90000
100000
80000
60000
40000
20000
0
Budget Expenditure
2015
2016
2017
2018
2019
DALY Injury
Budget Expenditure

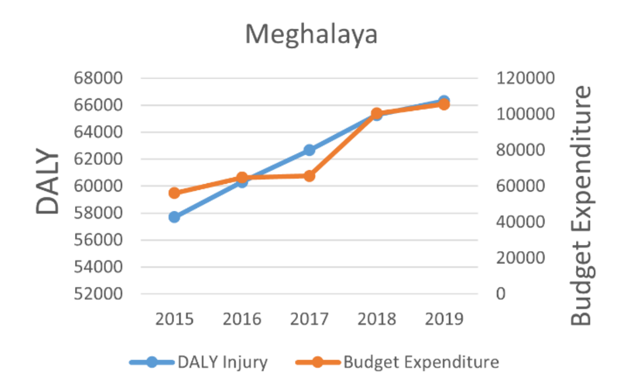
Meghalaya
DALY
68000
66000
64000
62000
60000
58000
56000
54000
52000
120000
100000
80000
60000
40000
20000
0
Budget Expenditure
2015
2016
2017
2018
2019
DALY Injury
Budget Expenditure

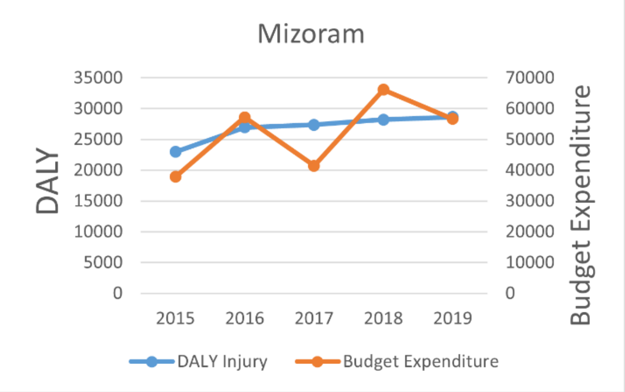
Mizoram
DALY
35000
30000
25000
20000
15000
10000
5000
0
70000
60000
50000
40000
30000
20000
10000
0
Budget Expenditure
2015
2016
2017
2018
2019
DALY Injury
Budget Expenditure

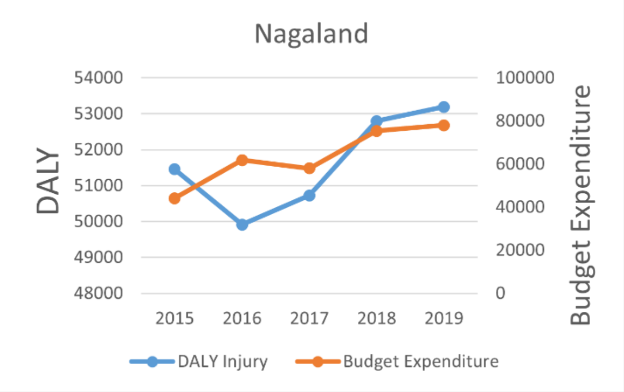
Nagaland
DALY
54000
53000
52000
51000
50000
49000
48000
100000
80000
60000
40000
20000
0
Budget Expenditure
2015
2016
2017
2018
2019
DALY Injury
Budget Expenditure

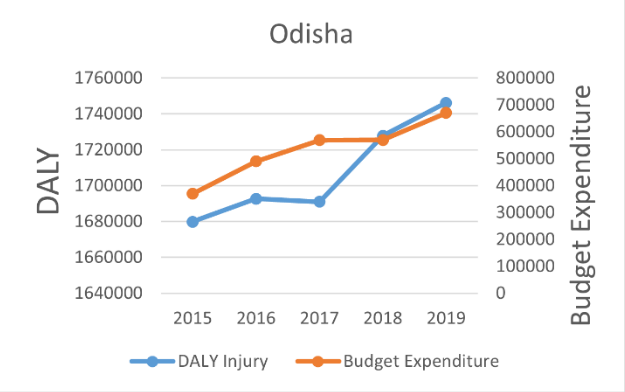
Odisha
DALY
1760000
1740000
1720000
1700000
1680000
1660000
1640000
800000
700000
600000
500000
400000
300000
200000
100000
0
Budget Expenditure
2015
2016
2017
2018
2019
DALY Injury
Budget Expenditure

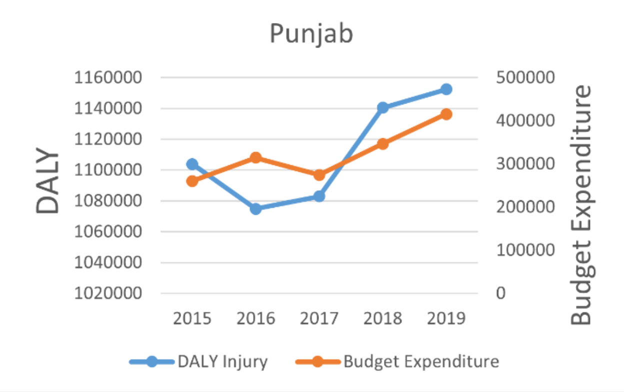
Punjab
DALY
1160000
1140000
1120000
1100000
1080000
1060000
1040000
1020000
500000
400000
300000
200000
100000
0
Budget Expenditure
2015
2016
2017
2018
2019
DALY Injury
Budget Expenditure

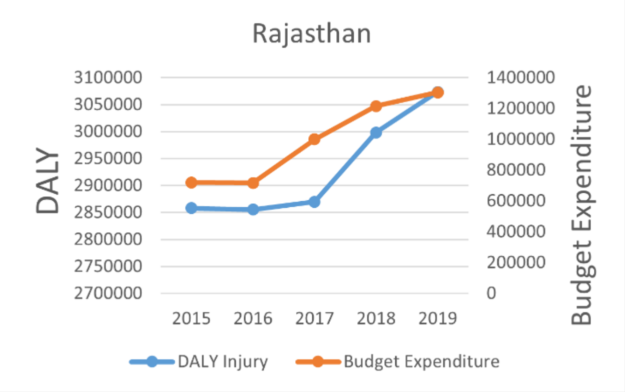
Rajasthan
DALY
3100000
3050000
3000000
2950000
2900000
2850000
2800000
2750000
2700000
1400000
1200000
1000000
800000
600000
400000
200000
0
Budget Expenditure
2015
2016
2017
2018
2019
DALY Injury
Budget Expenditure

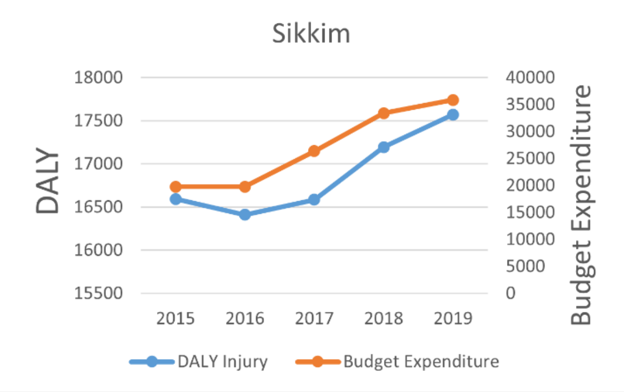
Sikkim
DALY
18000
17500
17000
16500
16000
15500
40000
35000
30000
25000
20000
15000
10000
5000
0
Budget Expenditure
2015
2016
2017
2018
2019
DALY Injury
Budget Expenditure

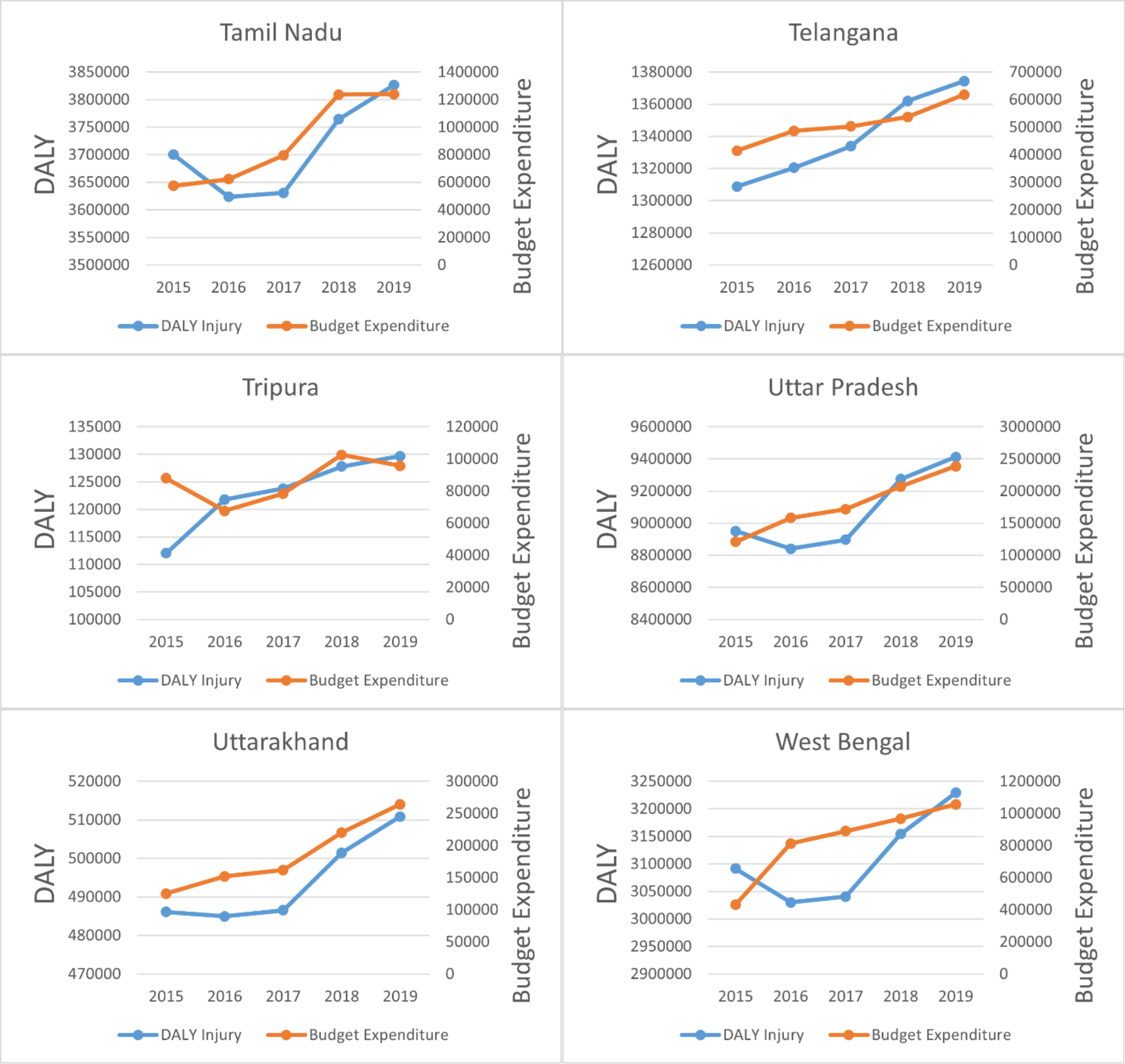

[Line graph with the primary Y-axis represents total DALY due to Injuries from 2015 to 2019 for individual states in India. Secondary Y-axis represents yearly budget during the same period for individual states in India]

Appendix VI. List of Diseases/health conditions used for the total disease burden calculation and analysis

**Communicable Diseases**	**Non-Communicable Diseases**	**Injuries/Accidents**
Acute hepatitis	Acne vulgaris	Adverse effects of medical treatment
African trypanosomiasis	Acute glomerulonephritis	Animal contact
Chagas disease	Age-related and other hearing loss	Conflict and terrorism
Cystic echinococcosis	Alcohol use disorders	Drowning
Invasive Non-typhoidal Salmonella (iNTS)	Alzheimer's disease and other dementias	Environmental heat and cold exposure
Dengue	Alopecia areata	Executions and police conflict
Diarrheal diseases	Anxiety disorders	Exposure to forces of nature
Dietary iron deficiency	Aortic aneurysm	Exposure to mechanical forces
Diphtheria	Appendicitis	Falls
Ebola	Asthma	Fire, heat, and hot substances
Encephalitis	Atrial fibrillation and flutter	Foreign body
Food-borne trematodiases	Attention-deficit/hyperactivity disorder	Interpersonal violence
Guinea worm disease	Autism spectrum disorders	Other transport injuries
HIV/AIDS	Bacterial skin diseases	Other unintentional injuries
Intestinal nematode infections	Bipolar disorder	Poisonings
Cysticercosis	Bladder cancer	Road injuries
Iodine deficiency	Blindness and vision loss	Self-harm
Leishmaniasis	Brain and central nervous system cancer	
Leprosy	Breast cancer	
Lower respiratory infections	Cardiomyopathy and myocarditis	
Lymphatic filariasis	Cervical cancer	
Malaria	Chronic kidney disease	
Maternal disorders	Chronic obstructive pulmonary disease	
Measles	Cirrhosis and other chronic liver diseases	
Meningitis	Colon and rectum cancer	
Neonatal disorders	Conduct disorder	
Onchocerciasis	Congenital birth defects	
Other intestinal infectious diseases	Decubitus ulcer	
Other neglected tropical diseases	Depressive disorders	
Other nutritional deficiencies	Dermatitis	
Other unspecified infectious diseases	Diabetes mellitus	
Otitis media	Drug use disorders	
Protein-energy malnutrition	Eating disorders	

Communicable Diseases	Non-Communicable Diseases	Injuries/Accidents
Rabies	Endocarditis	
Sexually transmitted infections excluding HIV	Endocrine, metabolic, blood, and immune disorders	
Schistosomiasis	Esophageal cancer	
Tetanus	Fungal skin diseases	
Trachoma	Gallbladder and biliary diseases	
Tuberculosis	Gallbladder and biliary tract cancer	
Typhoid and paratyphoid	Gout	
Upper respiratory infections	Gynecological diseases	
Varicella and herpes zoster	Headache disorders	
Vitamin A deficiency	Hemoglobinopathies and hemolytic anemias	
Whooping cough	Hodgkin lymphoma	
Yellow fever	Hypertensive heart disease	
Zika virus	Idiopathic developmental intellectual disability	
	Idiopathic epilepsy	
	Inflammatory bowel disease	
	Inguinal, femoral, and abdominal hernia	
	Interstitial lung disease and pulmonary sarcoidosis	
	Ischemic heart disease	
	Kidney cancer	
	Larynx cancer	
	Leukemia	
	Lip and oral cavity cancer	
	Liver cancer	
	Low back pain	
	Malignant skin melanoma	
	Mesothelioma	
	Motor neuron disease	
	Multiple myeloma	
	Multiple sclerosis	
	Nasopharynx cancer	
	Neck pain	
	Non-Hodgkin lymphoma	
	Non-melanoma skin cancer	
	Non-rheumatic valvular heart disease	
	Oral disorders	
	Osteoarthritis	
	Other cardiovascular and circulatory diseases	
	Other chronic respiratory diseases	
	Other digestive diseases	
	Other malignant neoplasms	

Communicable Diseases	Non-Communicable Diseases	Injuries/Accidents
	Other mental disorders	
	Other musculoskeletal disorders	
	Other neoplasms	
	Other neurological disorders	
	Other pharynx cancer	
	Other sense organ diseases	
	Other skin and subcutaneous diseases	
	Ovarian cancer	
	Pancreatic cancer	
	Pancreatitis	
	Paralytic ileus and intestinal obstruction	
	Parkinson's disease	
	Peripheral artery disease	
	Pneumoconiosis	
	Prostate cancer	
	Pruritus	
	Psoriasis	
	Rheumatic heart disease	
	Rheumatoid arthritis	
	Scabies	
	Schizophrenia	
	Stomach cancer	
	Stroke	
	Sudden infant death syndrome	
	Testicular cancer	
	Thyroid cancer	
	Tracheal, bronchus, and lung cancer	
	Upper digestive system diseases	
	Urinary diseases and male infertility	
	Urticaria	
	Uterine cancer	
	Vascular intestinal disorders	
	Viral skin diseases	
